# Senescent accelerated prone 8 (SAMP8) mice as a model of age dependent neuroinflammation

**DOI:** 10.1186/s12974-021-02104-3

**Published:** 2021-03-18

**Authors:** Andrés Fernández, Elena Quintana, Patricia Velasco, Belén Moreno-Jimenez, Belén de Andrés, Maria Luisa Gaspar, Isabel Liste, Marçal Vilar, Helena Mira, Eva Cano

**Affiliations:** 1 Chronic Disease Programme, Neuroinflammation Unit, Instituto de Salud Carlos III, Carretera Majadahonda-Pozuelo, Km.2,2, Majadahonda, 28220 Madrid, Spain; 2 Unidad de Inmunobiología, Instituto de Salud Carlos II, Madrid, Spain; 3 Instituto de Biomedicina de Valencia, Consejo Superior de Investigaciones Científicas, Madrid, Spain

**Keywords:** Aging, Brain myeloid cells, Microglia, Inflammation, IL-1β, SAMP8

## Abstract

**Background:**

Aging and age-related diseases are strong risk factors for the development of neurodegenerative diseases. Neuroinflammation (NIF), as the brain’s immune response, plays an important role in aged associated degeneration of central nervous system (CNS). There is a need for well characterized animal models that will allow the scientific community to understand and modulate this process.

**Methods:**

We have analyzed aging-phenotypical and inflammatory changes of brain myeloid cells (bMyC) in a senescent accelerated prone aged (SAMP8) mouse model, and compared with their senescence resistant control mice (SAMR1). We have performed morphometric methods to evaluate the architecture of cellular prolongations and determined the appearance of Iba1^+^ clustered cells with aging. To analyze specific constant brain areas, we have performed stereology measurements of Iba1^+^ cells in the hippocampal formation. We have isolated bMyC from brain parenchyma (BP) and choroid plexus plus meningeal membranes (m/Ch), and analyzed their response to systemic lipopolysaccharide (LPS)-driven inflammation.

**Results:**

Aged 10 months old SAMP8 mice present many of the hallmarks of aging-dependent neuroinflammation when compared with their SAMR1 control, i.e., increase of protein aggregates, presence of Iba1^+^ clusters, but not an increase in the number of Iba1^+^ cells. We have further observed an increase of main inflammatory mediator IL-1β, and an augment of border MHCII^+^Iba1^+^ cells. Isolated CD45^+^ bMyC from brain parenchyma (BP) and choroid plexus plus meningeal membranes (m/Ch) have been analyzed, showing that there is not a significant increase of CD45^+^ cells from the periphery. Our data support that aged-driven pro-inflammatory cytokine interleukin 1 beta (IL-1β) transcription is enhanced in CD45^+^BP cells. Furthermore, LPS-driven systemic inflammation produces inflammatory cytokines mainly in border bMyC, sensed to a lesser extent by the BP bMyC, showing that IL-1β expression is further augmented in aged SAMP8 compared to control SAMR1.

**Conclusion:**

Our data validate the SAMP8 model to study age-associated neuroinflammatory events, but careful controls for age and strain are required. These animals show morphological changes in their bMyC cell repertoires associated to age, corresponding to an increase in the production of pro-inflammatory cytokines such as IL-1β, which predispose the brain to an enhanced inflammatory response after LPS-systemic challenge.

**Supplementary Information:**

The online version contains supplementary material available at 10.1186/s12974-021-02104-3.

## Background

As life expectancy increases, age and age-related diseases have become a major health concern in western societies. Aging is the strongest risk factor for neurodegenerative diseases and while aging in itself is not considered a disease, results in important changes in brain tissue: significant increase in glial activation, complement factors and inflammatory mediators concomitant with brain atrophy [1, 2]. Microarray and single-cell RNAseq studies of aged human and mouse brains have further extended these findings by showing that genes related to cellular stress and inflammation increase with age, while genes related to synaptic function/transport, growth factors, and trophic support become downregulated [3–6].

A link between aging, neuroinflammation, and promotion of neurodegenerative diseases such as Alzheimer disease (AD), Parkinson disease, or vascular-associated dementia has been proposed [7]. Neuroinflammation (NIF), defined as the brain’s immune response, has been linked to age-associated neurodegeneration (reviewed in [8]). The central nervous system (CNS) was considered to be isolated and protected from immune responses, but it now appears that the blood–brain barrier (BBB) is not impermeable and that neuroinflammation occurs in the brain with similar features to inflammation in the periphery. In addition, a role for adaptive immune response in the CNS is now accepted (reviewed in [9, 10]). The main indicators of a primary neuroinflammatory response are phenotypic glial activation and de novo production of immune signaling molecules. Both, astrocytes and microglia, undergo cellular hypertrophy with increased expression of cell-surface immune modulatory proteins, including those of the major histocompatibility complex (MHC). These changes are accompanied by increased synthesis and release of pro-inflammatory cytokines and chemokines (reviewed in [11]). Although different types of cells have been involved in CNS inflammation, the main ones belong to the myeloid cell compartment. Brain myeloid cells (bMyC) mainly comprise microglia cells, and include perivascular, meningeal, and choroid plexus macrophages, periphery-derived monocytes, and brain dendritic cells (bDC), with a role in the establishment of neuroinflammation [12].

Parenchymal microglia, often referred as brain macrophages, are clearly implicated in the steady-state brain and in the response to different brain injuries. They have a myeloid origin; derive from embryonic yolk sac progenitors and are sustained thereafter by local progenitors [13–15]. One of the hallmarks of microglia cells is their age-dependent morphological changes. Microglia cells in the young healthy brain are cells with long and ramified prolongations [16, 17]. Aged microglia cells present ramified morphologies that are less branched, have shorter overall process lengths, and cover less dendritic “territory” than young microglia. Therefore, aged microglia presents a reduced “arborisation” [18, 19] and have characteristic accumulation in clusters, which might correspond to microglia responding to anomalous brain protein accumulation, stress related processes, or a direct effect of the aging process on microglia itself (reviewed in [1, 20, 21]). Furthermore, upon an inflammatory stimulus, these cells adopt an activated phenotype, which is characterized by morphological changes and cytokine synthesis, finally acquiring phagocytic capacity [22]. Morphologically, this activation is characterized by hypertrophy of cell body, widening of proximal processes and reduction of distal branches [23]. Therefore, the observation of changes in microglia cells in particular and bMyC in general, is a main characteristic of brain neuroinflammation.

Aging, as a progressive process associated to chronic low-grade inflammation and their neuroinflammatory-associated events, has been linked to neurodegenerative diseases [24]. In rodent models of Alzheimer’s disease (AD), chronic stress exacerbates neurodegeneration and cognitive impairments [25, 26], concomitant with increases of Aβ peptide accumulation and Tau protein phosphorylation (reviewed in [27]). In this context, the senescence accelerated mouse prone 8 (SAMP8) has been proposed as a neurodegeneration model to study late onset Alzheimer disease (LOAD) related to aging [20, 28–30]. This model presents many of the hallmarks found in neurodegenerative processes, as impairments in learning tasks, altered emotions, abnormality of the circadian rhythm [31], spongy degeneration [32], neuronal cell loss [33], and gliosis in the brain [34]. Notably, SAMP8 mice also show other characteristics seen in AD patients, such as memory deficits [35], increased Aβ levels [21], impaired neurogenesis [36, 37], activation of microglia, and an unfavorable inflammatory microenvironment [38, 39]*.* The neuroinflammatory state of SAMP8 model has been studied [40–42] but the specific changes that take place in the brain myeloid compartment of these animals remain to be fully explored.

The hallmark of neuroinflammatory response is phenotypic glial activation and the production of immune signaling molecules such as interleukin-1β (IL-1β). IL-1β is involved in elaboration of acute neuroinflammatory events, and the main cellular source in the brain is microglia (reviewed in [43]). Aged microglia cells are skewed toward a phenotype characterized by increased pro-inflammatory cytokine release, such as IL-1β, tumor necrosis factor-α (TNF-α), and IL-6 [19, 44]. Furthermore, augmented expression of these pro-inflammatory cytokines has been observed in SAMP8 brain tissues [42], and an increase in inflammatory markers has been described in SAMP8 blood plasma. Also, persistent microglial pro-inflammatory activation exacerbates neuronal damages and amyloidosis already present in AD pathology [45].

In this context, it has been described that systemic inflammation has a role in the progression of chronic neurodegenerative diseases. A hallmark of aged microglia is the fact that they are primed by chronic inflammatory environment and, therefore, aged microglia responds more vigorously to a given systemic inflammatory stimuli. This phenomenon was introduced as microglia priming [19, 46, 47]; reviewed in [48]. Innate immunity activates defensive mechanisms within minutes of microbial invasion through pattern recognition receptors (PRRs) such as Toll-like receptors (TLRs). TLRs have been involved in microglia responses to different insults, such as lipopolysaccharides (LPS), components of the outer membrane of Gram-negative bacteria or very different brain insults such as prion related peptides [49, 50].

LPS are responsible for the induction of inflammatory conditions, and LPS systemic administration has been widely used to test immune system activation and their role in neurodegeneration [51]. Although high LPS doses can lead to pathological reactions such as the induction of septic shock, low doses of circulating LPS are associated with chronic disease characterized by persistent low-grade inflammation [52]. This inflammatory response involves activation of PRRs and subsequent production of many pro-inflammatory cytokines such as IL-1β, TNF-α, or IL-6. In the present study, we have asked if isolated bMyC from aged senescent SAMP8 recapitulate this enhanced response.

In this work, we have analyzed the number and function of bMyC in the senescence SAMP8 model compared to the resistant control strain SAMR1. We have analyzed the morphology and distribution of hippocampal macrophages/microglia (Iba1^+^) cells by immunohistochemistry. We have quantified the number of hippocampus Iba1^+^ using stereology methods comparing young (2 months) with aged (10 months) animals from both strains, isolated and analyzed the number of CD45^+^ cells by flow cytometry studies. Finally, we have studied the expression of pro-inflammatory cytokines produced by isolated bMyC from the two main areas in which they are abundant: the BP and brain areas in contact with the periphery, m/Ch. We have further evaluated the response to low doses of inflammatory challenge on isolated cells from BP and m/Ch, and we have uncovered different activation kinetics of cells from both brains localizations.

## Methods

### Mice

Adult (2 to 10 months old) SAMP8 and SAMR1 mice were bred and maintained in the animal facilities at the Centro Nacional de Microbiología, Instituto de Salud Carlos III (CNM-ISCIII, Madrid, Spain). Male double transgenic APP/PS1 mice (10 months old), a cross between Tg2576 (overexpressing human APP695) and mutant PS1 (M146L), were kindly provided by Eva Carro [53]. All animal experiments were approved by the Institutional Review Board at the ISCIII and carried out in strict accordance with EU and National Animal Care guidelines. Protocols were approved by Consejería de Medio Ambiente Comunidad de Madrid (PROEX 179-14).

### Tissue processing

For confocal preparations, mice were deeply anesthetized by intraperitoneal (ip) injection of a mixture of ketamine and xylazine and transcardially perfused with 25–30 mL of saline solution for 5 min, followed by 10 min incubation with 4% paraformaldehyde (PFA, Sigma), pH 7.4, in 0.1 M phosphate buffer (PB, Sigma). After perfusion with the fixative, brains were dissected out and post fixed with 4% PFA for 18–20 h at 4°C. After fixation, brains were rinsed in 0.1 M PB and placed in 15% sucrose at 4°C until they sank, and then in 30% sucrose in PB at 4°C for 72 h. Finally, brains were embedded in tissue freezing medium (Tissue-Tek O.C.T^TM^, Sakura), by submerging them in increasing concentrations of OCT, frozen immediately in dry-ice-cooled 2-methylbutane (Sigma), and stored at −80°C. Coronal sections (30 μm) were cut using a CM1950 cryostat (Leica Microsystems). Brain sections were collected sequentially in 10 slides. Six sections per slide generate antero-posterior reconstructions of the hippocampus conformed by 1 section every 300 μm of hippocampal structure. Slides were stored at −20 °C until use.

### Periodic acid–Schiff (PAS) stain

Frozen brains embedded in OCT were cut into 30-μm thick sections as described before. Sections of the central zone of the hippocampus were selected according to mouse brain atlas [54]. Brain sections were dried at RT for 24 h, and sequentially submerged in absolute ethanol (EtOH) (2x), in 96% EtOH (2x), and in 70% EtOH (1x). Finally, samples were hydrated in deionized water and immerse in 0.5% periodic acid for 5 min at room temperature (18–26 °C). Slides were rinsed in several changes of distilled water and immersed in Schiff’s Reagent (Sigma) for 15 to 20 min at 4 °C. Slides were rinsed in running tap water for 5 min. For nuclei staining, slides were counterstained in hematoxylin solution, Gill No. 3 (Thermo Fisher), for 10 min, rinsed with alcohol acid (0.5% HCl in EtOH) three times and a final rinsed in tap water. Finally, slices were dehydrated, cleared, and mounted in DPX (Sigma) media.

### Immunohistochemistry

Immunohistochemistry analyses were performed on frozen brain sections by standard indirect staining as in [55]. Antibodies were diluted in 0.1 M PB containing 1% fetal bovine serum (FBS, Hyclone), 0.06% Triton-X100 (Sigma), and 150 mM glycine (Merck). Rabbit anti-Iba1 (1:100, Wako) was used to detect expression of bMyC; rat anti-mouse I-A/I-E (1:100, clone 2G9, B.D. Pharmingen) was used to detect antigen-presenting cells; mouse anti-phospho-Ser 139-Histone H2AX (clon JBW301, Millipore) was used to analyze DNA damage. Alexa fluor 488-labeled donkey anti-rabbit, -rat, -mouse, and Cy3-labeled donkey anti-rat antibodies (Jackson) were used as secondary antibodies. After staining, all sections and cells were mounted and preserved with 50% Mowiol (Polysciences), 2.5% DABCO (Sigma).

### Confocal microscopy and analysis

Images were acquired on a Leica Spectral SP5 confocal microscope. Brain maps were imaged using a ×20 immersion objective and a 1.7 digital zoom. Tissue images are tiles of 2-4 μm z-stacks and cell images are single 1-2 μm z stacks both captured with 40x and 63x oil objectives. Images are presented as average projections of z-stacks and keeping parameters constant. Exceptions are mentioned in figure legends. Negative control slides stained with primary antibody were used to identify potential nonspecific, background fluorescence. Acquired z-stacks were background-subtracted with the Leica LAS AF 2.6.3 software and secondary processed and analyzed using Adobe Photoshop CS3 (Adobe Systems) and ImageJ (National Institute of Health, http://rsb.info.nih.gov/ij) for ROI quantification and cell counting.

### Stereology and statistical analysis

Stereology was performed by the analysis of 6 coronal sections, 30 μm each, and separated 300 μm one from each other. Sampling started at first appearance of the infrapyramidal blade of the dentate gyrus (DG). Antero-posterior Bregma coordinates of all 6 sections correspond approximately to −1.2 mm to −2.7 mm [54]. Every quantification was normalized with the DG area of every section.

For statistical analysis, the GraphPad Prism software (Version 8.4.1; GraphPad Software, San Diego, CA 92108) was used after testing the normality of the data distributions with the Kolmogorov-Smirnov and D’Agostino-Pearson tests. Analysis of variance (ANOVA) was used for statistical analysis of differences between ages of the same strain. Post-hoc comparisons were performed using the Tukey test, and the Bonferroni correction was applied. Data were also analyzed by unpaired two tailed Student’s *t* test for comparisons of both SAMR1 and SAMP8 strains of the same age. Data are presented as mean ± standard error of the mean (SEM) and *n* indicates the number of independent mice used per strain and age. A *p* value of <0.05 was considered as statistically significant.

### Image analysis for morphometric parameters calculation

Three-dimensional (*3*D) reconstruction of individual microglial cells z-stack confocal images of around 30 μm thickness at intervals of 1 μm were taken at specified areas of SAMR1 and SAMP8 brains of different age. 3D images were obtained by using the plugin 3D viewer of the FIJI software (freely downloadable from http://fiji.sc/Fiji). To analyze spatial coverage of microglia, we used modified methods as described in [18]. Briefly, brain sections from these mice were stained with Iba1, hippocampal formation (Hpp) together with areas within: strata pyramidale (sp) and oriens (so) were imaged with confocal microscopy as before. Then, gray-level maximum z projection images were set to eliminate background based on intensity threshold and converted to binary images, processed with the “skeletonize” option in the FIJI software, and further analyzed with a modified Sholl’s analysis adapted for microglia as in [18, 56]. A representative heat-map image was generated based on 8-bit z-projection image using the 3D Surface Plot plug-in bundled in the FIJI software. For morphometric parameter calculation, after tracing an individual microglial cell, we calculated the number of intersections between microglial processes and concentric circles originated from the center of individual cell by using the Sholl’s analysis plug-in (Ghosh Lab, UCSD, San Diego, CA, USA) bundled in FIJI. This was used to analyze the number of intersections in circles with a radius between 5 μm (starting radius) as minimal distance corresponding to the soma of the cell and the final radio which includes the longest microglia branch (ending radius). We analyzed at least 25 individual Iba1^+^ cells from at least three individual animals. We present the data by using polynomial regression and defining three parameters: critical value of the circle radius (which defines the place of a possible circle intersecting maximum number of dendrites); the maximum number of ramification intersections with the circles (counted for consecutive circles placed starting at the cell body to the border of the arborisation and the mean value of the fitted polynomial function (which describes an average property concerning number of branches of ramification tree over the whole region occupied by the ramification arbor). For that purpose, we also used the Sholl regression coefficient as well as the Schoenen ramification index similar to that described previously [57, 58].

### LPS administration to mice

To assess the response of brain myeloid cells to peripheral immune stimulation, mice received a single intraperitoneal injection (i.p.) of 0.5 to 1 mg/kg LPS from E. coli, #0111:B4; L4391-IMG (Sigma). The LPS powder was dissolved in 0.9% endotoxin-free sterile saline at a concentration of 10 mg/mL. Mice injected with sterile saline as vehicle were used as control. Brains were collected and cell preparations and mRNA expression analyses were as follow.

### Cell preparation

Two and 10 months old SAMP8 and SAMR1 mice (or otherwise specified) were sacrificed, and intracardiac perfusion was performed using phosphate buffered saline (PBS) with observed blanching of the spleen during 5 min at a speed of 5 mL/min. Complete brains were dissected and for most experiments, meningeal (piamater) membranes and choroid plexus (m/Ch) were carefully removed with fine tweezers. Brain tissue was finely minced into small pieces and treated with a specific protease mix depending on the tissue. For brain without m/Ch, cells were prepared as in [55]. Brain, without the m/Ch and cerebellum, was digested in 5 mL of enzyme solution 20 units/mL papain (Worthington) and 0.025 units/mL DNase (Sigma) in buffer containing 116 mM NaCl, 5.4 mM KCl, 26 mM NaHCO_3_, 1 mM NaH_2_PO_4_, 1.5 mM CaCl_2_, 1 mM MgSO4, 0.5 mM EDTA, 25 mM glucose, and 1 mM l-cysteine, pH 7.5 for 30 min at room temperature (RT) with agitation. The brain homogenate was washed and filtered once through a 70-μM filter to remove undigested fragments and then washed twice more, followed by centrifugation at 300×*g* for 7 min. Cells were resuspended in 30% Percoll (GE Lifesciences) under 5 mL Hanks’ Balanced Salt Solution (HBSS) and centrifuged at 300×*g* for 20 min at RT with slow acceleration and no brake. Pellets were collected and washed with ice-cold PBS containing 2% (vol/vol) FBS (spin 300×*g*, 7 min). All subsequent washes were performed in this buffer. This preparation contains microglia/macrophages from brain as in [59], with the exception of those from meningeal membrane, choroid plexus, and cerebellum; we refer to this cell preparation as brain myeloid cells (bMyC) from BP. For m/Ch cell isolates, meningeal membranes plus choroid plexus were collected in an Eppendorf containing 1 mL PBS. Tissue was treated with 2.5 mg/mL Pronase (Roche) plus 0.025 units/mL DNase (Sigma) in PBS during 30 min at 37°C in a bath with mixing. This was followed by homogenization with gentle trituration using glass pipettes. m/Ch homogenates were filtered and treated as above. All subsequent steps were performed at 4°C in PBS, 2% FBS.

### CD45^+^ cell purification

After enrichment on Percoll gradient, brain isolated cells were purified using MACS LS columns from Miltenyi Biotec, following manufacturer’s instructions. Cells were washed in PBS 2% FBS and resuspended in 180 μL of PBS 0.5% BSA 2mM EDTA per brain sample, using MACS solutions. All steps were carried out on this buffer. CD45 mouse microbeads (20 μL) were added to the cells and incubated on ice during 15 min. Five milliliters were then added and cell suspension was centrifuged 10 min at 300×*g*. Supernatant was discarded and cells were resuspended in 5 mL and applied to the column, attached to MACS Separator and previously equilibrated with same buffer. Before passing the preparation through the column, cell preparation was filtered with 70-μm filter. The column was washed with 10 mL MACS solution and cells were extracted from column in 5 mL according to Miltenyi protocol. Cells were centrifuged again and resuspended in 1 mL of 2% FBS in PBS, counted in a Neubauer chamber and prepared for following applications.

### Flow cytometry

Single-cell suspensions were prepared as above and resuspended in staining buffer (2% FBS in PBS). Nonspecific binding to Fcγ receptors was blocked with 10 μg/mL of 2.4G2 mAb (Fc block, BD Biosciences). Staining was performed following standard protocols. Fluorochrome-labeled antibodies specific for mouse CD45, CD11b, P2RY12, and CD49d were from BD Biosciences or Biolegend. Cells were analyzed on a LRS Fortessa X-20 (BD Biosciences) cytometer, using the FlowJo v6.3.4 (TreeStar) and DIVA v8.0 software packages (BD Biosciences). The gating strategy used to exclude dead cells and doublets as in Additional Fig 2s.

### RNA isolation and real-time PCR

Total RNA was isolated with TriPure Isolation Reagent (Roche). Two micrograms of RNA were reverse transcribed using MMLV Transcriptase Reverse (Invitrogen), following the manufacturer’s instructions, except for using, 600 ng of random primers and 4 units of RNase OUT (Invitrogen).

Relative amounts of cDNA were analyzed by quantitative real-time PCR (qRT-PCR) using the FAST SYBR-green system and 7500 thermal cycler (Applied Biosystems). Ten nanograms of cDNA were analyzed in 15 μL reaction volume that includes 7.5 μL of 2x Reaction Mix, 0.5 μL of 10 μM of each primer. Thermal protocol consisted of a denaturation step at 95°C for 20 s, then 40 cycles of 3 s at 95°C and 30 s at 60°C, followed by a final step of 15 s at 95°C and 1 min at 60°C. Finally, a melting curve was performed from 60 to 95°C with 1% temperature ramp rate. Mouse 36b4 expression was used as an internal control to normalize for variations in input RNA. The amount of target mRNA in samples was estimated following the 2^−ΔΔCT^ relative quantification method [60].

Used primers are as follows:

**Table:** 

36b4:	Forward: AGATGCAGCAGATCCGCAT
	Reverse: GTTCTTGCCCATCAGCACC
Il-1β:	Forward: CAACCAACAAGTGATATTCTCCATG
	Reverse: GATCCACACTCTCCAGCTGCA
Tnf-α:	Forward: TGGAACTGGCAGAAGAG
	Reverse: CCATAGAACTGATGAGAGG
Il-6:	Forward: GAGGATACCACTCCCAACAGACC
	Reverse: AAGTGCATCATCGTTGTTCATACA
Ccl2:	Forward: CGGAACCAAATGAGATCAGAACCTAC
	Reverse: GCTTCAGATTTACGGGTCAACTTCAC
Aif-1:	Forward: GGGAAAGTCAGCCAGTCCT
	Reverse: GCATCACTTCCACATCAGCTT
Cx3cr1:	Forward: TCAGCATCGACCGGTACCTT
	Reverse: CTGCACTGTCCGGTTGTTCA

## Results

### Hippocampal PAS positive granules increase in aged SAMP8

**Fig. 1:**
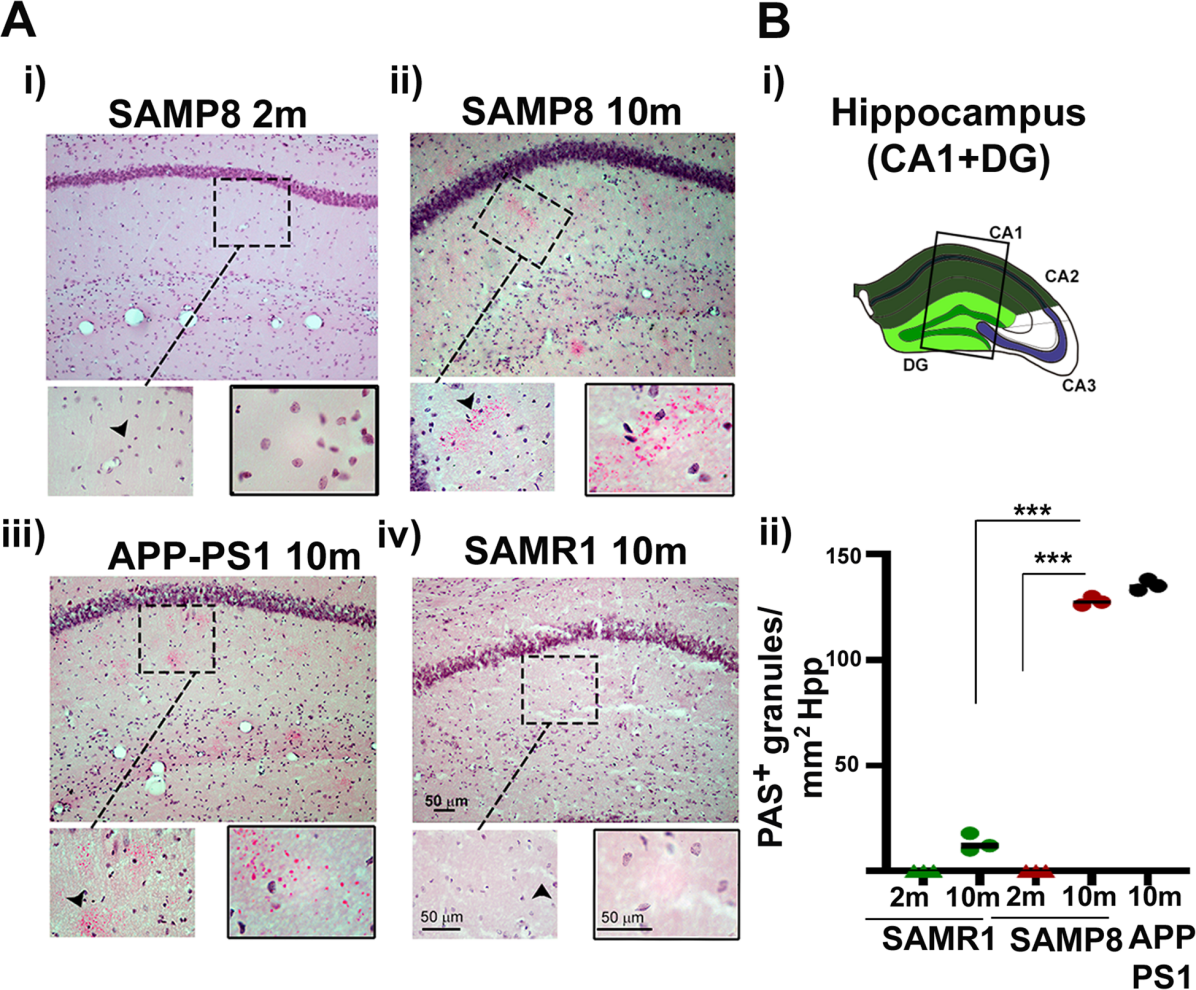
Analysis of PAS accumulations in SAM mice associated with aging and neurodegeneration. (**a**) Representative captures of hippocampal regions in SAMR1 and SAMP8 mice of 2 and 10 months (m) and 10 m old APP-PS1 mice, showing PAS-positive granules. Brain sections were stained by Schiff’s reagent (red-pink) and nuclei with hematoxylin (blue-brown). Images of coronal sections of cryostat (30 μm thick) were obtained with a ×20 objective (upper images), ×40 and ×100 (from left to right for magnified pictures), with a Nikon Eclipse 50i H550S light microscope and processed with the help of the NIS elements of software and ImageJ. (**b**) (i) Schematic representation of the regions analyzed. (ii) Quantification of PAS positive accumulates/mm^2^. SAMR1 is shown in green, SAMP8 in red, and APP PS1 in black. Two months data are represented with triangles and 10 months with circles. **p < 0.01 and ***p < 0.001 with respect to the PAS/mm^2^ accumulations (*n* = 3 to 5 males). Scale bars are included in the images

As it has been previously stated, the presence of granular accumulation is a hallmark of the neurodegenerative associated process in human brain [61], and aged animals [62]. These accumulations are often referred to as PAS positive granules, although their nature in different mouse strains is still under discussion. PAS positive granules have been observed in the hippocampus and entorhinal cortex of a variety of different species and associated to aging in SAMP8 animal model (reviewed in [63]). To localize a valid area of the brain in which neurodegenerative-associated processes are taking place, we analyzed 2 months and aged 10 months old animals from the SAMP8 strain. We also evaluated 10 m old SAMR1 Hpp as non-senescent control, and aged 10 m transgenic APP-PS1 Hpp as neurodegeneration-associated control. PAS positive granules were observed in hippocampus (Hpp) mainly in the CA1 region as described [64] and shown in Fig. 1a. The hippocampus (Hpp) in this study refers to the *dentate gyrus* (DG) and the *cornu ammonis 1* (CA1) area (scheme Fig. 1b, panel i). As shown in Fig. 1b, panel ii, a very clear increase of PAS positive granules was observed in Hpp from 10 m old SAMP8 brains, compared to 10 m SAMR1, in numbers that were comparable with those obtained in 10 months old APP-PS1 transgenic animals (Fig. 1b). The presence of these aged-associated protein accumulations indicate that brain Hpp is a suitable area to study the neurodegeneration associated neuroinflammation derived events that take place.

### Iba1^+^ cells from aged SAMP8 mice present clear morphological changes

**Fig. 2:**
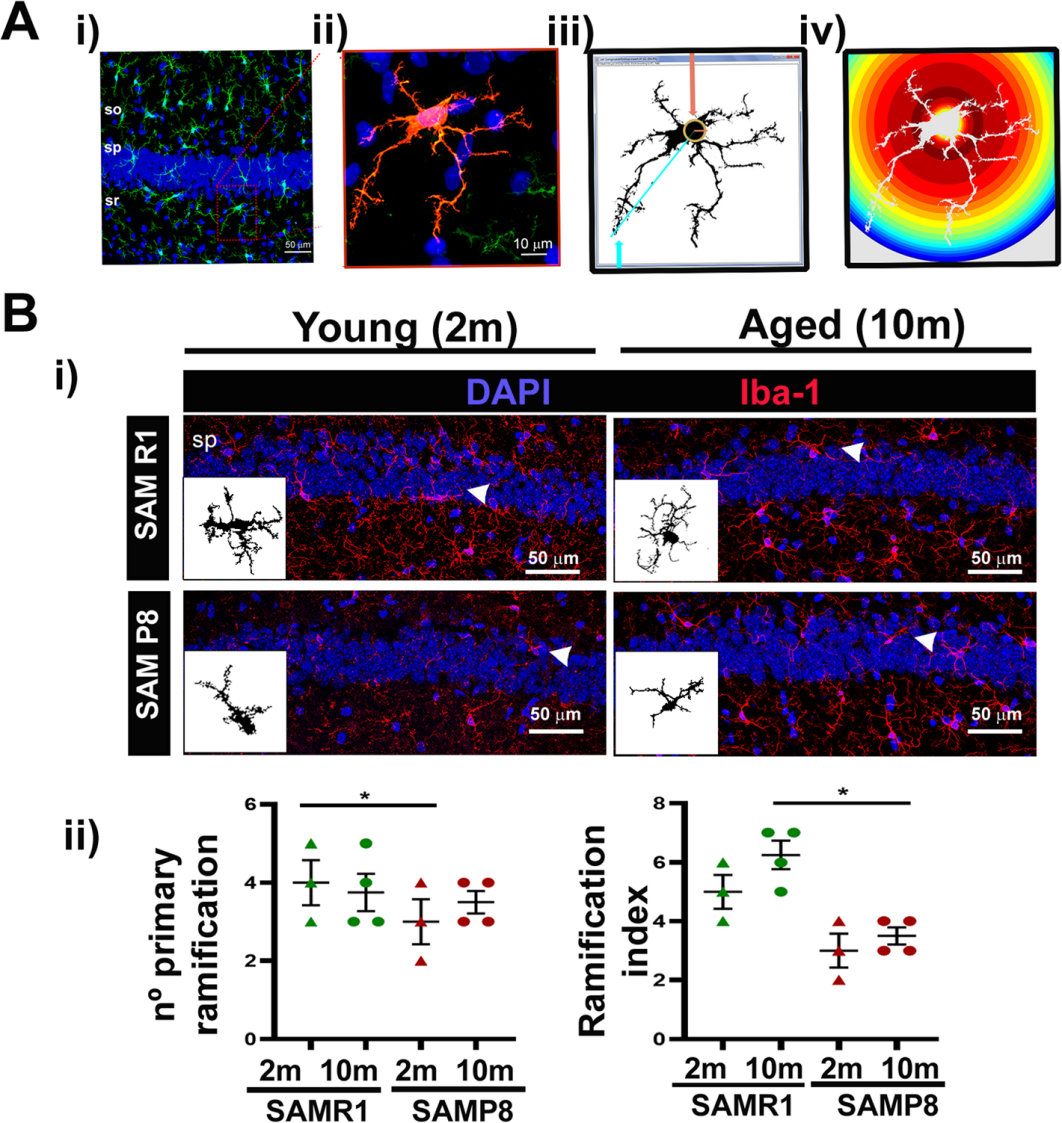
Morphology analysis by Sholl analysis. (**a**) (i) Representative images of hippocampus Iba1^+^ (green) regions used for analysis. so, stratum oriens; sp, stratum pyramidale; sr, stratum radiale. (ii) Detailed capture of cell morphology of an Iba1^+^ cell (red) maximum projection. (iii) Binary image of the maximum projection of the detailed image. (iv) Cartoon showing the radius of the longest extension that corresponds to the radius of the largest concentric circle and furthest from the soma (ending radius or maximum radius) and the radius that would cover the soma (starting radius or initial radius). (**b**) (i) Representative images of brain maps from 2 months and 10 months SAM Hpp areas using a ×40 objective and ×1.7 digital zoom. Insets show ×63 detailed images of iba1^+^ positive binary images for cell morphology. Nuclei are stained with DAPI (blue); brain microglia/macrophages are stained with Iba-1 (red). Scale bars are included in the images. (ii) Sholl analysis results, number of primary branches and branch index from Iba1^+^ cells from 2 (triangles) and 10 months (circles) old SAM mice Hpp area (*n* = 3 male). SAMR1 is shown in green and SAMP8 in red. Images were obtained with a Leica SP5 TCS inverted fluorescence confocal microscope, from 30 μm thick cryostat sections

Microglial morphology is altered in the cortex of aged human [2] and old mice [18]. Therefore, we aimed to study the complexity of microglia processes with age in SAMP8. We took serial z-stack and traced the backbone of cells throughout them with the FIJI software, as specified in material and methods. We performed Sholl analysis as a morphometric method that evaluates the architecture of cellular prolongations; these analyses draw a series of concentric circles around the cell soma, and consist of a mathematical method that gives a measure of the prolongation arborisation that can be used to study microglia ramifications [65, 66]. We obtained the number of primary ramifications as the number of extension originated in the cellular body or soma, and the Schoenen ramification index (SRI), described as the ratio between the maximum number of the intersections of microglia ramifications with the circles and the number of the primary ramifications. Our analyses show that 2 months old SAMP8 Hpp presented a lesser arborisation index than their 2 months old Hpp SAMR1 controls. In contrast, 10 months old SAMP8 Hpp Iba1^+^ cells have similar number of primary ramifications compared to their 10 months SAMR1 counterparts, although significant changes in their ramification index were observed (Fig. 2). Therefore, we conclude that Iba1^+^ cells in aged SAMP8 animals present morphological changes similar to those described in normal aging [18, 67]. Furthermore, aged SAMP8 Iba1^+^ cells occupied smaller territories, and they displayed more irregular processes than control animals.

### Clustered Iba1^+^ cells in aged SAMP8 do not express specific markers for DNA damage (γH2AX) nor activation related marker such as MHCII

**Fig. 3:**
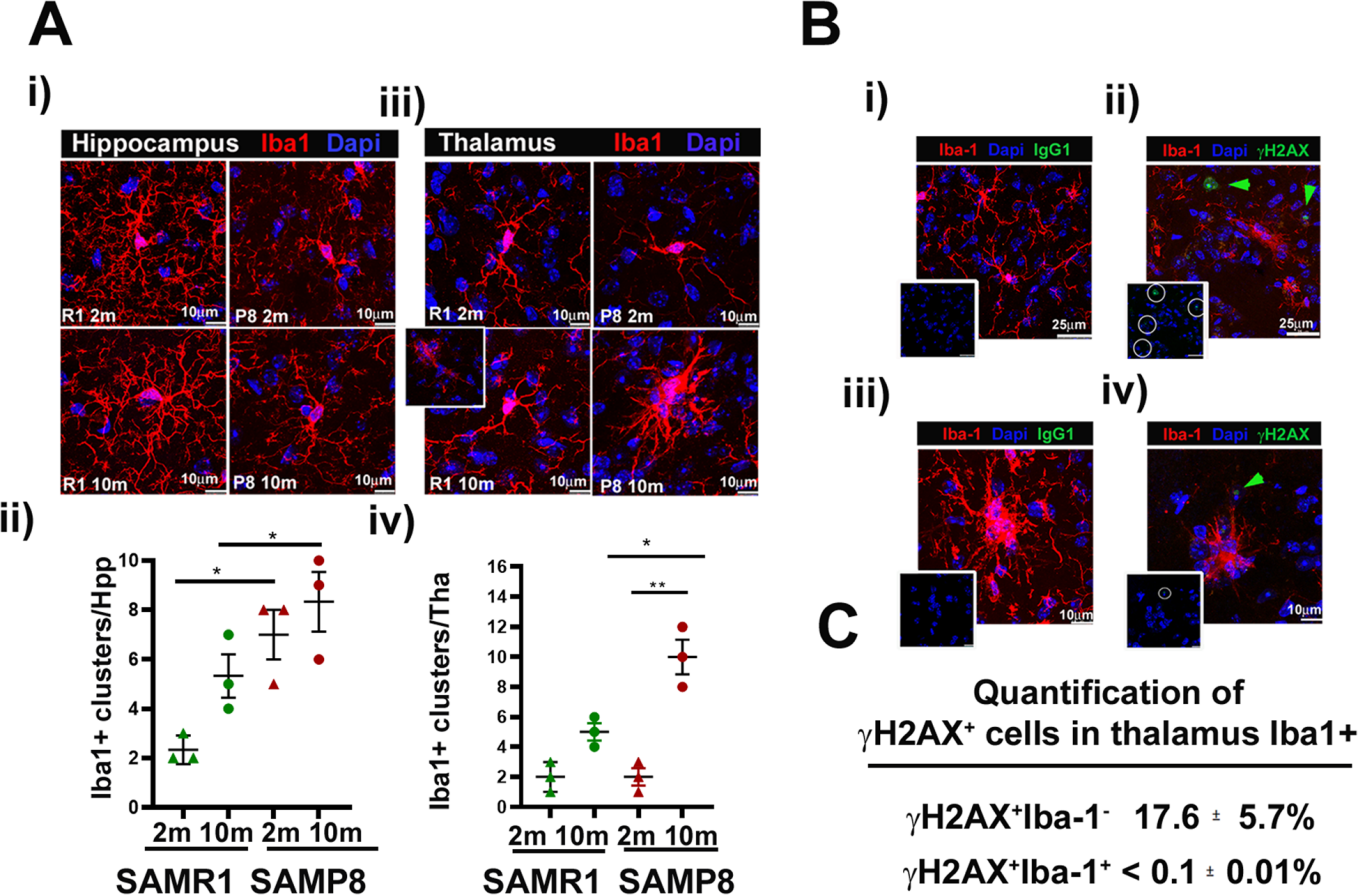
Iba1^+^ clusters analysis of young and aged SAM mice. (**a**) 2 and 10 months SAMR1 and SAMP8 brains were stained with Iba1 for microglia/macrophages (red) and DAPI for nuclei (blue). Images from Iba1^+^ stained brain tissue are maximal projection of 15-20 μm z-stacks obtained on a Leica Spectral SP5 confocal microscope with a ×63 oil objective (×3 digital zoom) to capture cellular arborisation. (i) Representative maximal projections of Hpp Iba1^+^ cells of young 2 months and old 10 months from SAMR1 as control and SAMP8 brains. Clustered cells of 2 to 3 nuclei were observed in SAMP8 animals at both age points. (ii) Quantification of hippocampus Iba1^+^ microglia clusters in SAMR1 and SAMP8 from 2- and 10-month-old animals. (iii) Representative maximal projections of thalamus Iba1^+^ cells from SAMR1 (in green) as control and SAMP8 (in red) brains from 2 months (triangles) and old 10 months (circles) old animals. Ten months old SAMP8 thalamic region presented many Iba1^+^ clustered cells. Insert images show small Iba1+ clusters found in 10m SAMR1 Hpp. (iv) Quantification of thalamus Iba1^+^ microglia clusters in SAMR1 (green) and SAMP8 (red) from 2 (represented with triangles) and 10 months (circles) old animals. Data in plots are values of Iba1^+^ clusters found in individual brains (*n* = 3) (**b**) Co-localization of Iba1^+^ immunofluorescence (red) and γH2AX^+^ (green) in 30 μm coronal cryostat sections of 10 m old SAMP8 brains. Parallel slides were stained with specific γH2AX^+^ followed by 488 IgG-anti-mouse antibody (panels ii and iv) or 488-IgG alone (panels i and iii). Panels (i) to (iv) show representative images of maximum projection as before, from 10 months SAMP8 thalamus. Panel (i) and (ii), images were taken with a ×63 oil objective (×1.9 digital zoom) to show a wide field, and panel (iii) and (iv) were taken with ×63 oil objective (×3 digital zoom) to show Iba1^+^ clusters. Iba1^+^ cells (red), DAPI (blue) and (panel i and iii) 488 IgG (green) and (panel ii and iv) γH2AX^+^ (green). The green arrows in panels ii and iv point to γH2AX^+^ cells outside Iba1^+^ clusters are marked with a green arrow showing antibody specificity. Inserts show DAPI (blue) and 488 (green) channels. Images are representative of an experiment of *n* = 3 males. (**c**) Quantification of γH2AX^+^ cells in Iba1^+^ thalamus of 10 months old SAMP8 animals. γH2AX^+^ Iba1^−^ cells are abundant in 10 months SAMP8 brains showing specificity of antibody. *p < 0.05, **p < 0.01, and ***p < 0.001; (*n* = 5) with respect to the number of clusters/mouse brain (*n* = 5 males). Scale bars are included in the images

Another age-dependent microglia morphological change is the appearance of dystrophic microglia and cellular clusters [68, 69]. Therefore, we analyzed the number of very apparent Iba1^+^ clusters (defined as multinucleated or joined cells) in SAMP8 and SAMR1 brains at different age. We chose two representative brain areas: the stratum oriens in the Hpp and the thalamus. Iba1^+^ clusters were hardly present in 2 m and 10 m SAMR1, whereas small clusters of 2 to 3 nuclei were observed in 2 months and 10 months old SAMP8 Hpp (Fig. 3a, panel i and quantified in panel ii). The number of these clustered cells was higher in SAMP8 Hpp versus SAMR1 Hpp independently of age (Fig. 3a, panel ii). When the thalamus region was visualized in these animals, the presence of big Iba1^+^ clusters was prominent in 10 months old SAMP8 brains (Fig. 3a, panel iii), although we found rare small clusters in thalamic 10 months SAMR1 brains regions (see insert and Fig. 3a, panel iv). The origin of these cellular clusters is unknown, and we hypothesize that might be due to cellular aging and associated DNA damage. DNA damage, either endogenous or exogenous, forms double stranded breaks (DSBs) a phenomena that is followed by the phosphorylation of the histone gamma H2AX (γH2AX^+^) [70]. Furthermore, expression of γH2AX^+^ cells has been used as a cellular senescence marker [71]. We evaluated the number of γH2AX^+^ cells in 10 months old SAMP8 animals, focusing on Iba1^+^ cells. We found γH2AX^+^ cells in aged 10 months SAMP8 brains, but most of these cells did not express the Iba1 marker, in accordance to previously reported data [72]. The number of double positive γH2AX^+^ Iba1^+^ cells in the thalamic regions, where Iba1^+^ clusters were most noticed, was very low (< of 0.1± 0.01%). These results indicate that clustered Iba1^+^ cells do not specifically present associated DNA damage events, and may be functional microglia. γH2AX^+^ cells that are not microglia are possibly astrocytes and neurons, which have previously shown to be positive in neurodegenerative brains [72–75].

Microglia cells represent the main antigen-presenting cells inside the BP during neurodegeneration. While expression of MHCII is low under homeostatic conditions in the brain, it can be rapidly upregulated on bMyC and it is used as a marker of their activation [76]. We asked if Iba1^+^ clusters observed in 10 months SAMP8 brains were in fact activated microglia described as Iba1^+^ MHCII^+^ cells. Our analyses showed that these Iba1^+^ clusters were negative for MHCII. The lack of staining was not due to technical issues, since we observed a good staining of MHCII^+^ cells in choroid plexus. In fact, we could detect a clear increase of Iba1^+^ MHCII^+^ cells staining in the choroid plexus of aged SAMP8 compared with young 2 months SAMP8 brains. Therefore, our studies indicate that thalamic Iba1^+^ clusters are not enriched in MHCII^+^ cells in aged SAMP8 BP (Additional Fig 1S).

### The number of hippocampal Iba1^+^ cells differs between aged senescent and control mouse strains

**Fig. 4:**
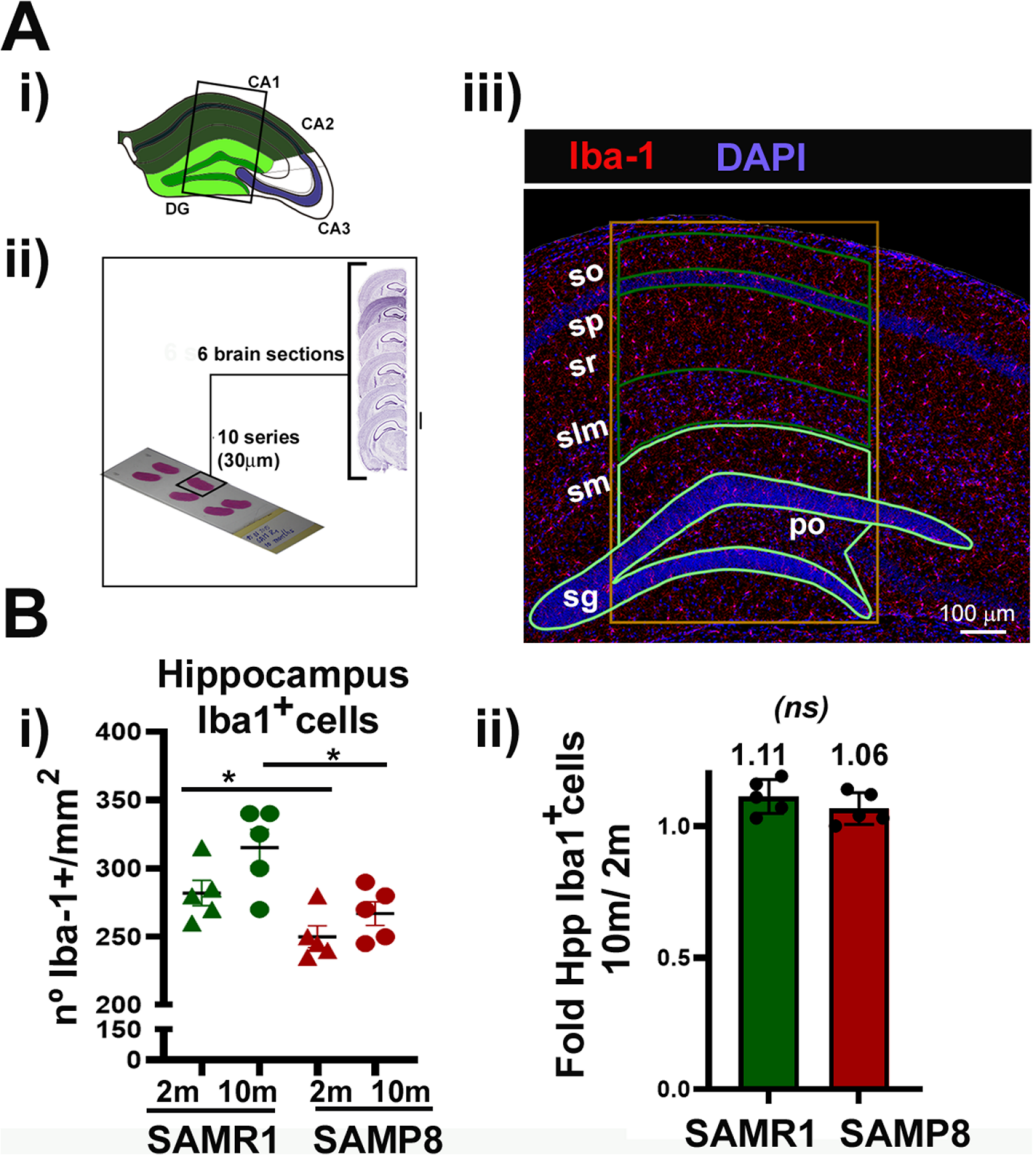
Evaluation of Iba1^+^ cells in the hippocampus of SAM mice aged 2 and 10 months. Thirty micrometers brain coronal cryostat sections obtained as in material and methods were stained with Iba1 (red) and nuclei with DAPI (blue). (**a**) (i) Hpp area used in the quantification of Iba1^+^ are surrounded by a box, differing in CA1 (cornus ammonis, dark green) and DG (dentate gyrus, soft green), as shown in the scheme. (ii) Scheme of sample collection: six coronal sections, 30 μm each, separated 300 μm one to each other were collected and processed. Sampling started at first appearance of the infrapyramidal blade of the dentate gyrus (DG) from approximately Bregma −1.2 mm to −2.7 mm. (iii) Representative map of the hippocampus (Hpp) showing the expression of BP Iba1^+^ myeloid cells Iba1^+^ in 10 m SAMR1 mice and indicating the regions used for counting. Images at ×40 (×1.7 digital zoom) were obtained with a Leica SP5 TCS inverted fluorescence confocal microscope and the areas were processed and evaluated with ImageJ. stratum oriens (so); stratum pyramidale (sp); stratum radiatum (sr); stratum lacunosum-moleculare (sm); stratum moleculare (sm), granule cell layer (sg); polymorph layer (po). Scale bars are included in the image. (**b**) (i) Quantification of Iba1^+^ cells in the area described in 2 months (triangles) and 10 months (circles) SAM mice. Data are number on Iba1^+^ cells per individual Hpp (*n* = 5 male). (ii) Fold change of Hpp Iba1^+^ of 10 months versus 2 months in SAMR1 and SAMP8 mice. *p < 0.05; **p < 0.01, and ***p < 0.001 with respect to Iba-1 +/mm^2^ cells in Hpp from SAMR1 2m (*n* = 5 males). In (i) and (ii) SAMR1 data are shown in green and SAMP8 in red

It has been described that the aging process might affect the number of Iba1^+^ cells in the brain [77] and particularly in the Hpp. These analyses have been performed as well in aged SAMP8 mice, in which an increase in CD11b staining has been described in this area [78]. We have analyzed if our animals recapitulate this characteristic feature. To focus on a specific brain area that would allow us to better compare the different strains and age samples, we chose to perform stereology measurements in the Hpp as specified in material and methods. Briefly, we performed analyses of 6 coronal sections separated 300 μm one to each other from young (2 months) and elderly animals (10 months) as in Fig. 4a, panel ii. These coronal sections correspond approximately to Bregma −1.2 to −2.7 mm from [54] reference atlas. Coronal brain slices were stained with anti-Iba1 antibodies and number of Iba1^+^ cells were evaluated. Hpp: DG and CA1 areas, as specified in Fig. 4a panel iii, were analyzed. Surprisingly, but very reproducible, immunohistochemical analyses of total Iba1^+^ cells in 2 months SAMP8 Hpp showed a significant reduction of Iba1^+^ cells compared to 2 months old SAMR1 control brains. This decrease in Iba1^+^ cells was further observed in 10 months old SAMP8 brains, when compared with their 10 months SAMR1 controls (Fig. 4b, panel i). Therefore, differences between strains complicated further comparisons of aged SAMP8 mice and SAMR1 mice. To eliminate the “strain factor,” we evaluated and compared fold increase of Iba1^+^ cells of aged (10 months) versus (2 months) in each of the strains. As shown in Fig. 4b, panel ii, our results in Hpp (CA1+DG), indicate that there are not clear differences in Iba1^+^ cells with age, even in 10 months old SAMP8 animals that show clear phenotypical changes associated to age reviewed in [20].

**Fig. 5:**
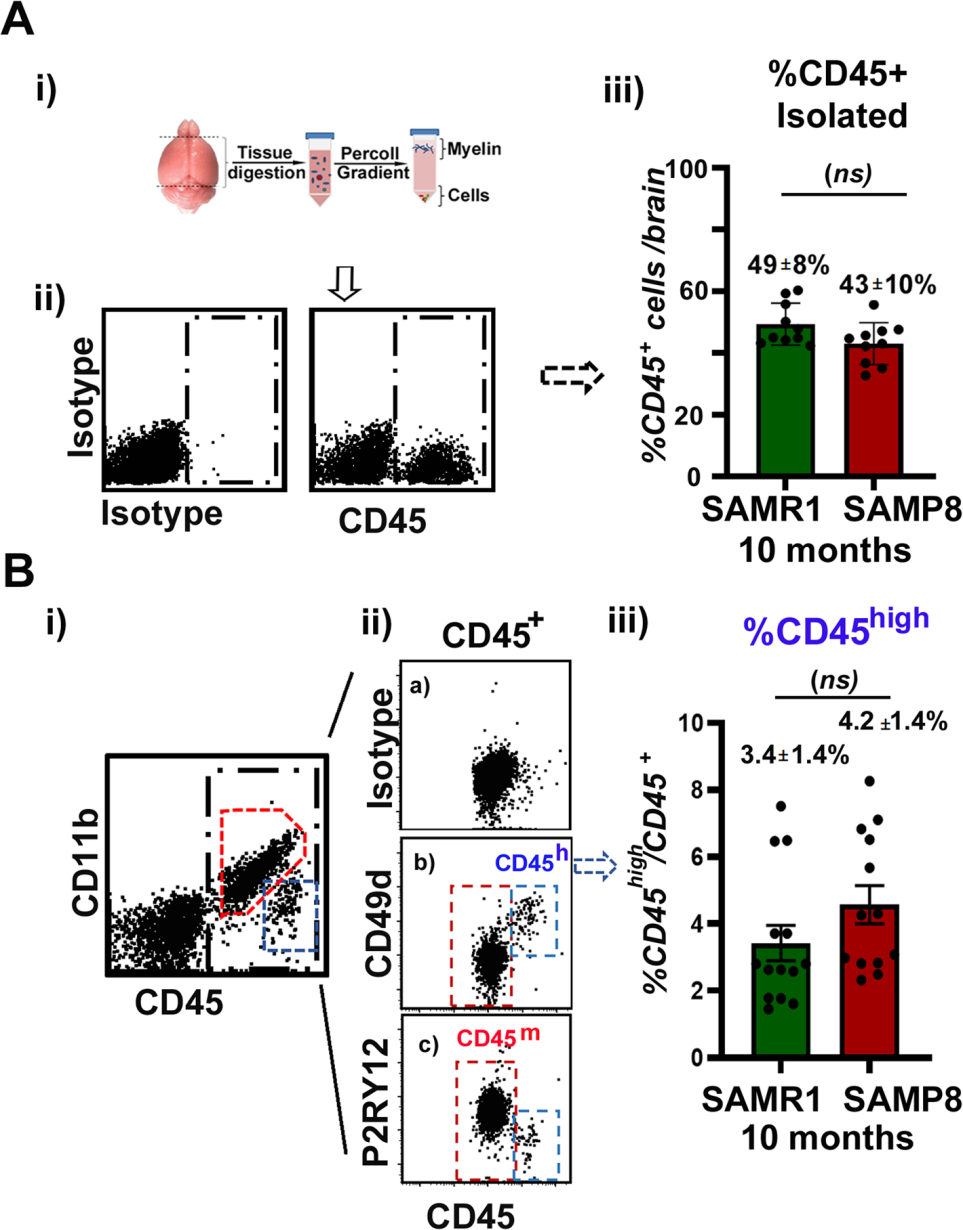
Isolation and quantification of CD45-positive brain cells. (**a**) (i) Cartoon representing brain cells isolation procedure. (ii) Representative flow cytometry dot plots of live cells isolated from brain (except cerebellum and olfactory bulb (OB) and m/Ch). Cells were isolated and stained with control isotype antibody (APC-labeled rat IgG2b) or with APC-labeled rat anti-mouse CD45. Black box marks CD45^+^ cells. Gating strategy was as presented in additional Fig 2s. (iii) The box and whisker plot shows arithmetic mean ± 25-75 quartile (box) and minimum and maximal values (lines) (*n* = 10) of the percentage of total CD45^+^ cells isolated from 10 months SAMR1 (green) and SAMP8 (red) brains. (**b**) (i) Cells were stained with PE-Cy7-labeled rat anti-mouse CD45 together with 488 rat anti-mouse CD11b. CD45^m^ cells are marked with red box and CD45^h^ cells with a blue box. (ii) Cells were stained with PE-Cy7 rat anti-mouse CD45 plus APC rat IgG2b as isotype control (**a**); APC rat anti-mouse CD49d (**b**) or APC rat anti-mouse P2RY12 (**c**). Shown are representative dot plots obtained for cells isolated from 10 months SAMP8 animals that were similar to cell preparations from young and old SAMR1 and SAMP8 animals. (iii) Box and whisker plot as in panel A iii (*n* = 10), representing the percentage of total CD45^h^ cells isolated from 10 months SAMR1 (green) and SAMP8 (red) brains. (*ns*) Indicates no statistically difference between specified groups applying statistical methods as in material and methods

A way to assess the number of bMyC present in the brain is by isolating brain cells followed by flow cytometry analyses of the different existing cellular populations. bMyC are the major player in inflammation and they are characterized by the expression of the pan-hematopoietic marker CD45 and the myeloid marker CD11b (see introduction). We isolated 2 months and 10 months old SAMP8 and control SAMR1 brain parenchymal cells without the meningeal and plexus membranes (BP) cells by using 30% Percoll gradient. The amount of alive cells obtained after this procedure was not statistically different between animals groups. For aged animals, we obtained 1.2 ± 0.1 million cells/brain from 10 months SAMR1 animals and 1.1 ± 0.1 million cells/brain from 10 months old SAMP8. Once those cells were we follow the gating strategy presented in Additional Fig 2s to eliminate doublets and further analyzed DAPI positive, alive cells. In Fig. 5a, panels i and ii, we present the number of CD45^+^ cells isolated per brain in 10 months aged SAMR1 (green bars) and SAMP8 (red bars). The number of isolated CD45^+^ bMyC did not significantly change between all the groups analyzed, and particularly in 10 months aged SAMR1 (49 ± 8%) and SAMP8 (43 ± 10%) *n* = 10 (Fig. 5a, panel iii). There is a clear discrepancy about the difference observed between Iba1^+^ immunohistochemical analysis (Fig. 4) and CD45^+^ cells cytometry analyses (Fig. 5a). To clarify this point, we evaluated the expression of Iba1 mRNA transcripts in both strains at the different age analyzed and observed that in fact, there is a clear decrease in the expression of *Aif-1* gene encoding Iba1 in 2 months SAMP8 animals, in accordance with the results obtained in our immunofluorescence analyses (Fig. 4). To assess if there were less Iba1^+^ cells, we evaluated the expression of a different molecule, that has been previously shown to be expressed mainly in microglia cells in the brain, the fractalkine receptor (CX3CR1) [79]. In this case, the amount of specific *Cx3cr1* transcript present in all the groups analyzed differed only slightly (Additional Fig 3s). This indicates that the number of BP microglia cells do not change greatly although there is a clear decrease in the expression of Iba 1 gen (*Aif-1*), which should be further studied. Since we have not found significant aged-associated changes in the number of Iba1^+^ between aged 10 months SAMR1 and SAMP8 by immunofluorescence analyses (Fig. 4b, ii), together with the fact that CD45^+^ isolated cells from 10 months SAMP8 BP do not show significant differences compared to 10 months SAMR1 control (Fig. 5a, iii), we concluded that the number of BP bMyC in the SAMP8 brain do not change with age. Furthermore, we suggest that the isolation and evaluation of isolated CD45^+^ BP cells is the method of choice to analyze changes between different mice strains and conditions.

### The number of BP CD45^high^ cells do not increase in age senescent SAMP8 mice

Immunosurveillance takes place in the central nervous system (CNS) in spite of its specific anatomic features conferring a certain degree of isolation from the periphery [80]. The blood-brain barrier (BBB) prevents free trafficking across the brain vasculature but immune cells can cross the endothelium of post-capillary venules or reach the brain through the choroid plexus and the leptomeninges (mCh) mainly under inflammatory conditions. To analyze if there was an increase in the number of CD45^+^ from the periphery, we stained isolated cells from the different strains and ages with anti-CD45 and CD11b antibodies (Fig. 5b), and observed more clearly that BP CD45^+^ cells can be differentiated in two clear populations: one that express medium levels of CD45 (CD45^m^, red box), and cells with a high expression of CD45 (CD45^h^, blue box). We found that there were not significant differences between isolated cells comparing age and strains. As mentioned before, the existence of brain CD45^h^ positive cells has been correlated with the existence of peripheral CD45^+^ cells in the brain. Cerebral invasion of lymphocytes crucially depends on the interaction of the leukocyte very late antigen-4 (VLA-4) with vascular cell adhesion molecule-1 (VCAM-1) on endothelial cells [81, 82]. VLA-4 (integrin α4β1) is an integrin heterodimer consisting of an alpha chain (integrin α4 = CD49d) and a beta chain (CD29). Therefore, the expression of CD49d has been used to trace brain myeloid cells originated in the periphery [83]. CD45^h^ cells in our cellular BP preparation were CD3 negative (data not shown) but mainly positive for CD49d (Fig. 5b, panel ii b, blue box), and this marker did not stain CD45^m^ cells (red box). To ensure that CD45^m^ cells correspond to *bonafide* microglia, we stained CD45^+^ cells with specific microglia markers such as P2RY12 receptor. As shown in Fig. 5b, panel ii c), this marker stained the majority of CD45^m^ brain cells, corresponding to 92 ± 4% (*n* = 4) of P2RY12^+^ cells from total CD45^+^ BP cell isolated with not significant differences between age and strains analyzed. The quantification of CD45^h^ cells in aged animals, in which BBB breach might occur, did not show significant differences between 10 months SAMP8 BP (4.2 ± 1.4%, *n* = 4) compared to their 10 months SAMR1 control (3.4 ± 1.4%, *n* = 4). In our hands, the number of CD45^h^ in aged animals was comparable to that observed in young 2 months control strains [55]. These results are further discussed in the following section.

### Inflammatory profile in isolated brain myeloid cells

**Fig. 6:**
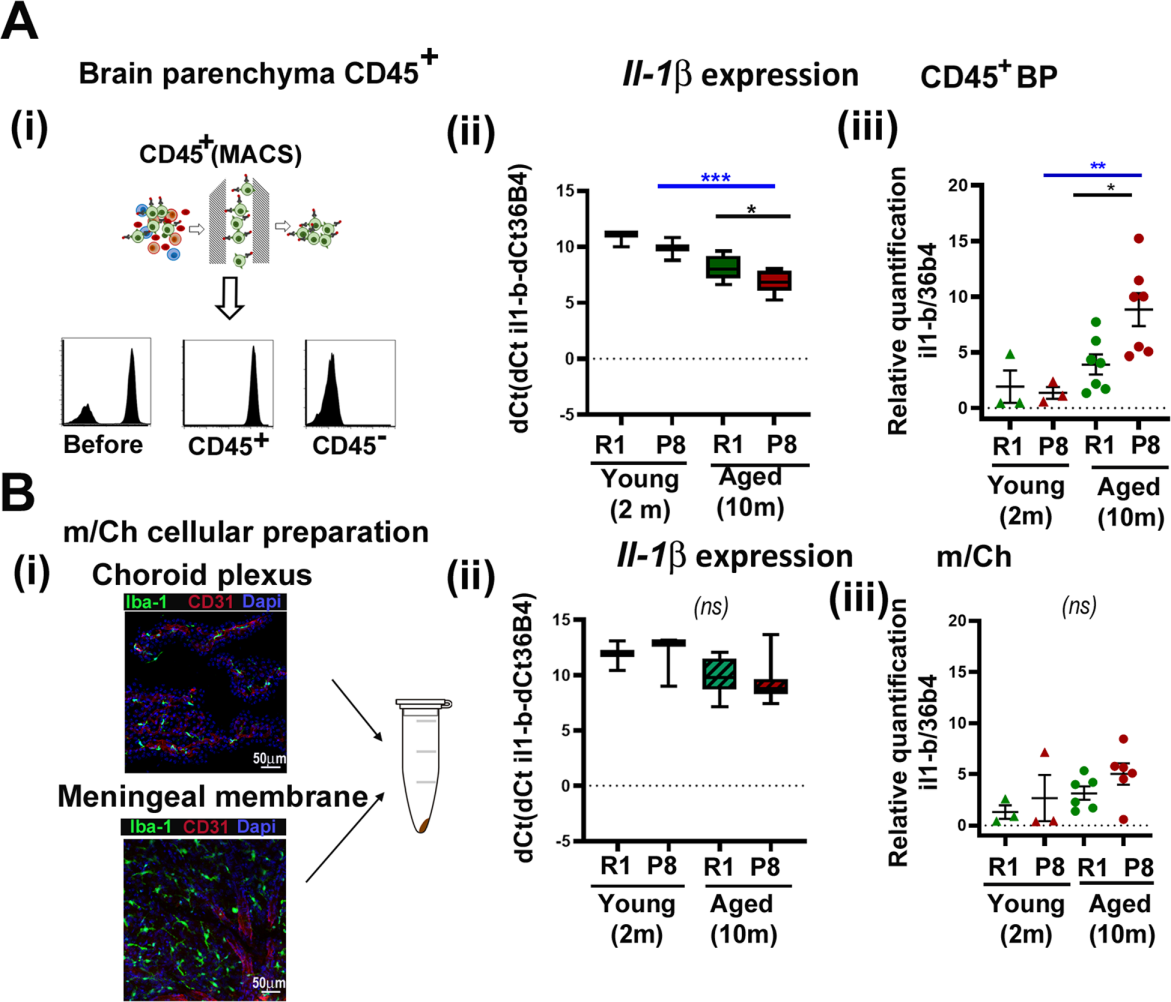
Higher *Il-1β* expression occurs mainly in aged SAMP8 CD45^+^ brain parenchymal cells. qRT-PCR analysis of *Il-1β* mRNA expression. (**a**) (i) Scheme of BP CD45 magnetic cell separation used for CD45^+^ isolation, and histograms (bottom) showing the CD45 staining by cytometry from CD45^+^ and CD45^−^ fraction. Quantification of BP CD45^+^ obtained in positive fraction that was of 97 ± 2% (arithmetic mean ± SEM (*n* = 5) of CD45^+^ cells from alive cells, determined as in additional Fig 2s. (ii) *Il-1β* mRNA expression from BP CD45^+^ isolated cells from young (2 months) and aged (10 months) SAMR1 (green) and SAMP8 (red) animals. Data in the box and whisker plot (as in Fig. 5a iii) are presented as dCt (Ct _il1β_-Ct _36b4_). Lower values of dCt means higher *Il-1β* transcript expression in the sample. (iii) Relative quantification of *Il-1β* mRNA expression was referred to average values of 2 months old SAMR1 as control animals that were given a value of 1. Plot represents individual values are mean ± SEM of *n* = 3 to 6 male brains. SAMR1 is shown in green and SAMP8 in red. Two months data are represented with triangles and 10 months data with circles. (**b**) (i) Dissected choroid plexus and meningeal membranes (m/Ch) were pooled into an Eppendorf tube and total RNA was obtained as indicated in material and methods. Specific brain regions were stained with anti CD31 for brain vessels (red), Iba1 (green) and DAPI for nuclei (blue), and representative images of these areas were taken with a SP5 Leica TCS confocal fluorescent microscope, show abundance of Iba1^+^ cells (green) by immunofluorescence in both brain localizations. Scale bars are included in the images. (ii) *Il-1β* mRNA expression from m/Ch preparations from young (2 months) and aged (10 months) SAMR1 (green) and SAMP8 (red) animals. Specific mRNAs were amplified from total mRNA qRT-PCR using 36b4 as reference gene. Data are presented as dCt (Ct _il1β_-Ct _36b4_) as in panel A (ii). Lower values of dCt means higher *Il-1β* transcript expression in the sample. (iii) As before, relative quantification of *Il-1β* mRNA expression was referred to average values of 2 months old SAMR1 m/Ch values as control animals that were given a value of 1. Two months data are represented with triangles and 10 months data with circles. SAMR1 is shown in green and SAMP8 in red. Plot represents individual values are mean ± SEM of *n*=3 to 6 male brains. **p < 0.01 and ***p < 0.001: (ns) means non-significant differences, *n* ≥ 3 male

As mentioned above, a characteristic of neuroinflammation is phenotypic glial activation and de novo production of immune signaling molecules. Both astrocytes and microglia undergo cellular hypertrophy with age, and it has been shown that pro-inflammatory cytokines levels increase in elderly subjects [18, 22, 84]. IL-1β is intimately involved in the elaboration of acute neuroinflammatory processes in vivo, and exposure of the rodent brain to IL-1β elicits rapid, robust activation of both astrocytes and microglia. In the senescent model SAMP8, an increase of inflammatory markers has been described in blood plasma and total brain tissue [42]. Therefore, we asked if a source of IL-1β in this model was in fact the bMyC cellular compartment, since they constitute the main cell type involved in the brain inflammatory process. We analyzed *Il-1β* expression in CD45^+^ bMyC preparations from BP. Strategy is summarized in Fig. 6a, panel i for BP, and from border-associated myeloid cells contained in m/Ch preparations as in Fig. 6b, panel i. *Il-1β* transcripts from 2 and 10 months old SAMP8 and SAMR1 cellular preparations were analyzed by qRT-PCR as in material and methods. We express our data in delta Ct (dCt) as the Ct of the specific transcript minus Ct of *36b4* as housekeeping control (Fig. 6a and b, panel ii), that more clearly shown the difference between very different tissues. As shown in Fig. 6, *Il-1β* mRNA expression was significantly higher in BP preparations from aged 10 months old SAMP8 when compared with 10 months old SAMR1. dCt data from m/Ch preparation were higher, indicating a lower abundancy of specified transcript. In fact, *Il-1β* transcripts were hardly detected in m/Ch when compared with BP cellular preparations. Figure 6a and b, panel iii shows the same data displayed as quantification relative to healthy 2 months SAMR1 animals that was given an average value of 1. Brain border (mCh) bMyC were analyzed to study the possible contribution of peripheral inflammation to brain *Il-1β* expression detected in aged SAMP8 BP (Fig. 6a).

A raise in IL-1β parenchymal expression in the rodent brain might increase the expression of other pro-inflammatory cytokines, leukocyte chemotactic chemokines, cell surface adhesion molecules, cyclooxygenases, and matrix metalloproteases within the brain parenchyma. We analyzed in our experimental groups the expression of pro-inflammatory cytokines such as tumor necrosis factor-alpha (TNF-α) and interleukin 6 (IL-6). Furthermore, IL-1β elevations have been implicated in trafficking of peripheral immune cells to the brain mediated by cytokines such as CCL2 (reviewed in [8]); therefore, we included the expression of this cytokine in our analyses. As shown in Additional Fig 4s, *Tnf-α*, *Il-6*, and *Ccl2* mRNA expression was significantly diminished in young (2 months) SAMP8 BP cellular preparations compared to 2 months old SAMR1, similarly to the data obtained for *Il-1β*. Although mRNA values of these cytokines were variable in 10 months old animals, an increase of *Il-1β* was observed in BP from SAMP8-aged animals (represented as a lower dCt: Ct_*gene*_- Ct_*36b4*_). When m/Ch preparations were analyzed, there were not significant changes in the expression of these markers between all the groups tested. We noticed that the expression of cytokines in m/Ch was very low for all the pro-inflammatory markers and lower than in BP preparations. Thus, these results indicate that aged SAMP8 bMyC express higher mRNAs levels of pro-inflammatory markers mainly in the brain parenchyma and not in border bMyC from m/Ch membranes. Consistent with these data, we have not detected a significant increase in systemic inflammation in 10 months SAMP8 animals, since our preliminary analyses do not detect an increase in inflammatory markers in blood plasma of these animals (data not shown).

### Response to inflammatory stimuli in the senescent model

The use of lipopolysaccharide (LPS) as a robust systemic inflammogen has been widely described (see introduction). For these experiments, we selected a low dose of LPS (i.p. injection 0.5 to 1mg/kg) that does not cause mouse lethality and only induces a limited inflammatory response [85, 86]. While high doses of LPS produce immune activation and lethality characteristic of septic shock, low doses such as the one selected in our studies reproduce the chronic inflammation status found in many neurological diseases. Furthermore, this compound has been used to test a phenomenon called “priming” of aged innate immune response (see introduction). We analyzed if old SAMP8 brains recapitulate this phenotype characteristic of brain aging.

**Fig. 7:**
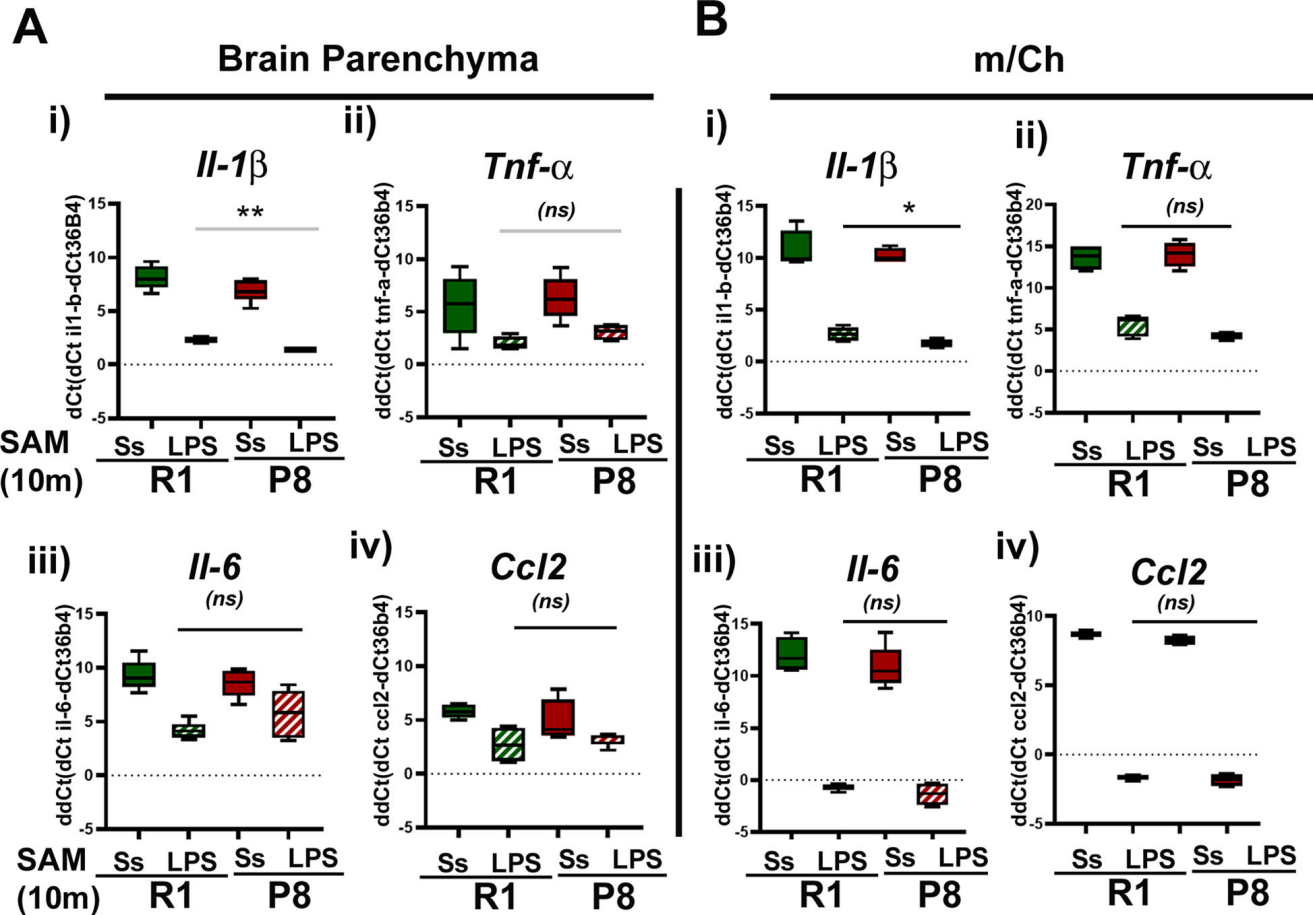
bMyC from brain parenchyma (BP) and m/Ch respond to low doses of systemic LPS. Isolated CD45^+^ cells were isolated form BP and m/Ch preparation as in Fig. 6. (**a**) BP CD45^+^ (**b**) m/Ch preparations, from aged (10 months) old SAMR1 (green bars) and SAMP8 (red bars) that were injected with saline (empty bars) or LPS (1 mg/mL) (stripped bars) for 3 h and *Il-1β* (panel i), *Tnf-α* (panel ii), *Il-6* (panel iii), and *Ccl2* (panel iv) mRNA expression was then qRT-PCR as before. Cytokines-specific mRNAs increased significantly 3 h after the injection of LPS. Data in box and whisker plots are presented as dCt (Ct _*Gene*_-Ct _*36b4*)_. (mean ±SEM) *n* = 3 males). Lower values of dCt means higher cytokine transcript expression in the sample. SAMR1 is shown in green and SAMP8 in red. Two months data are represented with triangles and 10 months with circles **p < 0.01 and ***p < 0.001. (ns) means non-significant differences between specified groups, *n* ≥ 3 males

First, we evoked systemic inflammation by LPS and analyzed the expression of *Il-1β*, *Tnf-α*, *Il-6*, *and Ccl2* mRNAs at different times, to define the time window for the expression of pro-inflammatory markers in our brain cellular preparation. In accordance with literature, after 3 h of LPS (1 mg/kg) intraperitoneal injection, brain tissue and isolated bMyC expressed high levels of all these pro-inflammatory markers [19, 87]. We evaluated the expression of these molecules in cellular preparations from BP and from border-associated m/Ch cells. As shown in Fig. 7a, expression of these molecules was greatly enhanced by LPS in BP, although maximal LPS-dependent transcription of these cytokines was observed in m/Ch border-associated cells, as shown in Fig. 7b. Expression kinetics were faster in m/Ch than in BP cells as correspond to a systemic model of infection (data not shown).

Once established the time and dose of the inflammogen, and the fact that systemic LPS was sensed by the BP cells inducing the expression of pro-inflammatory markers, we analyzed the inflammatory state in bMyC cell preparation as before. Senescent 10 months old SAMP8 and control SAMR1 were injected with saline solution (ss) and LPS (as in material and methods), and after 3 h, BP and m/Ch cells were isolated. Levels of inflammatory markers were analyzed as previously described. As shown in Fig. 7, LPS induced all the pro-inflammatory markers expression studied in both animal strains, represented as dCt: Ct_*gene*_- Ct_*36b4*_*.* It was noticeable that only the *Il-1β* expression was significantly higher in aged LPS-challenged SAMP8 animal compared to their control LPS-SAMR1. This phenomenon agrees with previous results in which there are an age-dependent priming of microglia from aged brains [19, 51], which we show is recapitulated in aged SAMP8 animals.

## Discussion

### SAMP8 as a model of neurodegeneration-associated neuroinflammation

Brain aging in mice and humans leads to morphological and functional changes that are considered hallmarks of abnormal activity. One of these morphological changes is the appearance of pathological granular structures in the Hpp and their progressive expansion with age [88]. These anomalous PAS positive aggregates have been described in aged SAMP8 mouse, and their nature has been discussed and reviewed in [61, 63]. Although SAMP8 animals have been proposed as a model for age-associated neurodegenerative diseases such as late onset Alzheimer disease (LOAD) (reviewed in [20]); the brain myeloid cells repertoire in the SAMP8 model has not been fully described. Early findings by Takeda’s group described the presence of accumulation of CD45^+^ CD11b^+^ cells by immunohistochemical analyses in the CA1 region of SAMP8 mice hippocampus [78], concluding that there was an increase in microglia proliferation and the appearance of reactive/activated microglia as cells with thick and short processes and strong staining of CD11b. The effect of aging alone in the number of microglia cells is controversial in the literature. Some authors do not observe an increase of microglia cells in male mice aged Hpp [89]; other authors found that the number of microglia cells is decreased in the aged hippocampal area [90, 91], or increased when female animals were analyzed [89]. These discrepancies might be due to different mouse strains, animal age or the fact that different markers for microglia detection and methods are used. A careful comparison of all these results awaits further analyses. In most of the studies, Iba1 staining has been used as a marker for microglia identity/activation, and Iba1 increased expression has been further reported in aged CA1 rat by immunoblot analyses [92]. Here, we have re-analyzed changes in the number and morphology of Iba1^+^ cells associated to senescent-related processes in the SAMP8 brain. Our results did not support an age-dependent increase of microglia cells in the aged SAMP8 brain by two ways. First, we performed stereology mapping to analyze the number of BP Iba1^+^ cells by immunofluorescence. These studies allowed us to compare more robustly different brain areas from a variety of mouse strains and age by measuring the expression of Iba1^+^ cells/mm^2^ in each strain. Second, the evaluation of the number of isolated CD45^+^ brain cells in aged SAMP8 compared to SAMR1 control (as shown in Fig. 5), allowed us to conclude that, at the age evaluated (10 m), there was not a significant increase of hippocampal Iba1^+^ cells in the old senescent animal SAMP8, with clear hallmarks compatible with brain degeneration. We clearly observed that young 2 months old SAMP8 presented less amount of Hpp Iba1^+^ staining when compared to their SAMR1 controls, which make difficult the comparison between strains. No fold increase differences between 2 and 10 months samples was observed (as in Fig. 4), and therefore we reached to the conclusion that the number of Iba1^+^ cells in the aged senescent mice with respect to their control do not increase. The meaning and final outcome of this reduction of Iba1^+^ staining in the young SAMP8 mice remains to be fully investigated.

One possibility to reconcile our observation to those previously reported, in which they observed an increase in the microglia number with age, could be the use of older animals (12 months old) that might account for the difference in number of Iba1^+^ cells observed, although not significantly differences at 10 months were observed (Fig. 4). Microglia cells increase, as CD11b^+^ cells, has also been described in response to SAMP8 aging, a process that is concomitant with age-dependent changes in BBB [93]. Nevertheless, we have not been able to detect such an increase, nor proliferation in the BP (Iba1^+^Ki67^+^ cells) by immunofluorescence (data not shown). Furthermore, we could not detect significant changes in the number of CD45^+^ isolated from these mice (Fig. 5a), nor a substantial increase in the amount of BP CD45^h^ cells from the periphery when compared 10 months old SAMP8 with their SAMR1 controls (Fig. 5b). These analyses are an experimental challenge since they required a careful manual dissection of meningeal membranes (piamater) and choroid plexus, which contained CD45^h^ cells. The novel description of cellular markers that differentiate proper CD45^+^ brain cells from those of peripheral origin will clearly help to confirm the permeability capacity of immune cells with aging.

The activation state of aged SAMP8 microglia can be studied by the expression of MHCII. MHCII expression is required to establish a robust adaptive immunity in the brain and periphery. The entry and activation of T cells in the brain parenchyma requires a pre-existing interaction of these cells with antigen presenting cells. In non-injured young brains, the expression of MHCII is low and mainly restricted to perivascular spaces and border associated macrophages in m/Ch [55, 94]. During the aging process, a strong increase in brain MHCII^+^ cells has been described in different animal models [92, 95–97]. We evaluated the presence of Iba1^+^ MHCII^+^ cells by immunofluorescence analyses in 10 months aged SAMP8 brains (Additional Fig 1s). MHCII^+^ cells in the BP of young 2 months SAMP8 were hardly detectable, and 10 months old SAMP8 animals did not express higher amount of Iba1^+^ MHCII^+^ cells in BP, mainly in thalamic areas, compared to their young 2 months SAMP8 control. This expression was very low, hardly detectable and restricted to perivascular areas, as seen in control animals [55]. To ensure that there were no technical problems with the immunofluorescence analyses, we studied the expression of MHCII antigen in the brain m/Ch border. We found a clear increase of Iba1^+^MHCII^+^ cells in m/Ch of 10 m SAMP8 compared to their 2 months controls. These experiments could not been performed in SAMR1 mice, since the antibody used for these analyses did not recognize the specific haplotype of the SAMR1 animals (H-2Ks) [98]. When 10 months aged control mouse strain such as CD1 was analyzed instead, we could not observe an increase in the number of Iba1^+^ MHCII^+^ cells in 10 months old animals, although the number of MHCII present in the m/Ch in young animals was higher (Additional Fig 1s). Age-dependent changes in the immune system and BBB function has been described and reviewed [99]. Our data show that MHCII^+^ Iba1^+^ cells are a minority in BP and the expression of this activation marker is mainly found in the Iba1^+^ cells of border m/Ch areas from aged SAMP8 mouse. Furthermore, this implies that the expression of this activation marker is not present in the clustered Iba1^+^ cells observed in aged SAMP8 mouse.

For the isolation of cells from two of the main bMyC enriched areas, BP and m/Ch [55], removal of myelin and cell debris after tissue digestion is a critical step for flow cytometry analysis. Percoll density gradients have been widely used for brain immune cells isolation [100, 101]. Different density gradients such as 30% [102–104] and 30-70% [19, 100, 105] have been used. In our hands, 30% Percoll density gradients produce more than threefold increase in the total cell recovery with a better viability. To achieve greater purity, we used magnetic-beads based affinity purification (MACS) with a yield of 97 ± 2% (*n*=10) CD45^+^ positive cells (cartoon in Fig. 6a). BP preparation will formally contain proper microglia cells, perivascular macrophages and blood-derived macrophages that might infiltrate the CNS and contribute to the pathological sequelae. The Hpp is an irrigated brain tissue, and since changes in BBB permeability have been reported in the SAMP8 [106, 107]; it is possible that peripheral myeloid cells enter the aged SAMP8 brain. As shown in Fig. 5, our BP preparations, in the most inflammatory conditions (10 months aged SAMP8 animals), contained mainly proper microglia cells with a medium CD45 expression as described before. Our results indicating that in aged SAMP8 mouse BBB permeability for peripheral lymphocytes was neither enhanced compared to aged control SAMR1 animals nor to young animals (Fig. 5), are in line with previous studies [108], although in contrast to the results described by other authors [106]. This topic has been recently reviewed in [99].

Increased levels of pro-inflammatory cytokines such as IL-1β, TNF-α, and IL-6 in the aged CNS has been described before [18, 19, 84, 109, 110]. Here, we have studied mRNA expression of these cytokines in bMyC-enriched preparation of SAMP8 and SAMR1 from BP and m/Ch with age. Our data in the aged senescent model are consistent with the increment of inflammatory markers seen in aged brain tissue and aged isolated brain.

Since IL-1β is intimately involved in elaboration of acute and chronic neuroinflammatory processes in vivo (revised in [43]); here, we show that *Il-1β* expression is greatly enhanced in aged SAMP8 bMyC from BP but not in m/Ch (Fig. 6). These results are in accordance to preliminary data indicating that systemic pro-inflammatory markers in aged 10 months SAMP8 animals do not differ greatly from 2 months or their 10 months SAMR1 control (data not shown). Parenchymal expression of *Il-1β* in rodents increases expression of other pro-inflammatory cytokines and leukocyte chemotactic chemokines within the brain parenchyma [43, 111]. Importantly, IL-1β is capable of triggering further increases in its own expression as evidenced by murine *Il-1β* induction following human IL-1β administration or expression in the brain [43, 112]. Therefore, an increment on the amount of IL-1β expression will lead to a neuroinflammatory cascade that it is restricted to the brain tissue in the absence of systemic challenges.

We have evaluated further bMyC response to low LPS dose simulating mild inflammatory conditions and not septic shock. The LPS dose used in our experiments (0.5 to 1 mg/kg) is far from the LD_50_ for LPS in mice (5 to 15 mg/kg) and did not cause significant lethality [85, 86]. Despite this low dose, BP bMyC were able to sense this inflammogen and produced LPS-dependent cytokine increase, indicating that the BBB-protected microglia cells are able to sense systemic inflammatory conditions. These results are in accordance to recent data indicating that the entire brain exhibited the ability to respond to endotoxemia provoked by LPS producing a wide repertoire of cytokines [113]. How and which are the receptors involved in LPS-inducible inflammation in the brain remains to be fully understood, although a role for TLR2 receptor has been suggested [50].

Aging dependent BBB permeability allows the entrance of peripheral immune cells in the SAMP8 model in response to peripheral inflammation. This phenomenon could play a role in the amplification of neuroinflammation processes in response to systemic inflammation and how to manipulate this entrance will be the focus of future research effort.

## Conclusions

Neuroinflammation (NIF) plays an important role in aged associated degeneration of central nervous system (CNS). Here, we analyzed the brain myeloid cells repertoire in the senescent accelerated prone aged (SAMP8) mouse model and propose this model to study age-associated neuroinflammatory events. Although aged hippocampal areas from SAMP8 animals do not show differences in the number of Iba1^+^ cells or with age, there are clear phenotypical changes associated to aging and an increment of bMyC inflammatory markers, together with a role in the amplification of the neuroinflammation processes in response to systemic inflammation. The need of experimental models to analyze the role of neuroinflammatory processes in the neurodegeneration associated to aging, opens the possibility to study the response of SAMP8 to novel therapeutic approaches.

## Supplementary Information

**Additional file 1: Supplementary Fig 1S.** Evaluation of Iba1^+^ MHCII^+^ cells in the choroid plexus of 2 and 10 months old SAMP8 and CD1 mice. Representative Iba1 (red), MHCII (green) and DAPI (blue) staining images of coronal sections of choroid plexus (A) from 2 months (triangles) and 10 months (circles) old SAMP8 in red (B) and 2 months and 10 months CD1 in black. Images were obtained with a Leica TCS SP5 inverted fluorescence confocal microscope, using a 40x and 63x objective (3x digital zoom) for the magnifications. The images are representative of at least three independent experiments using males. Scale bars are included in the images. Panels ii) show analysis of the number of Iba1^+^ MHCII^+^ cells over the total of Iba1^+^ cells. The data show the mean ± SEM (n = 3 to 4 male). * *p* < 0.05 ; (*ns*) no significant differences between groups.

**Additional file 2: Supplementary Fig 2S**. Gating strategy for flow cytometry analysis. Cells were obtained and labeled as described in Materials and Methods. (A) Brain parenchyma cells without m/Ch (B) m/Ch isolated cells. Before flow cytometry analysis DAPI (5 μg/mL) was added to determine cell viability. Cells were first gated (P1) based on size and complexity (SSC vs FSC). Doublet discrimination was performed by FSC-H vs FSC-W and SSC-H vs SSC-W analyses, and then PI or DAPI negative cells (i.e. live cells) were selected for further analysis.

**Additional file 3: Supplementary Fig 3S.** *Aif-1 and Cx3cr1* in CD45^+^ brain parenchymal cells. Isolation and quantification of BP CD45^+^ brain cells as in Fig 6. qRT-PCR analysis of (A) *Aif-1* gene and (B) *Cx3cr1.* After extraction of total RNA, cDNA were amplified using SYBR Green Real time PCR methodology using *36b4* as reference gene. (i) Show graphs presented as dCt (Ct _gene_-Ct _*36b4*_). Lower values of dCt means higher cytokine transcript expression in the sample. (ii) Relative quantification of mRNA expression was referred to average values of 2 m old SAMR1 as control animals that was given a value of 1. The data show the mean ± SEM (n = 3 to 4 males). ** *p* < 0.01 between specified groups, n ≥ 3 males.

**Additional file 4: Supplementary Fig 4S**. *Tnf-α, Il-6 and Ccl2* expression occurs mainly in aged SAMP8 CD45^+^ brain parenchymal cells. qRT-PCR analysis of (i) *Tnf-α*, (ii) *Il-6* and (iii) *Ccl2* mRNA expression was then quantified by real-time PCR as before. (A) CD45^+^ brain parenchymal (BP) cells as Fig 6. (B) Dissected choroid plexus and meningeal membranes (m/Ch) were obtained as in Fig 6b. After extraction of total RNA, cDNA were amplified as described in Fig 3s. Data are presented as dCt (Ct _gene_-Ct _36B4_). Lower values of dCt means higher transcript expression of specific mRNA in the sample. The data show the mean ± SEM (n= 3 to 4 males). ** *p* < 0.01 and *** *p* < 0.001 between specified groups.

## Data Availability

The datasets generated for this study are available on request to the corresponding author.

## Abbreviations

CNS: Central nervous system
SAMP8: Senescent accelerated prone
SAMR1: Senescence resistant
NIF: Neuroinflammation
AD: Alzheimer disease
LOAD: Late onset Alzheimer disease
bMyC: Brain myeloid cells
BP: Brain parenchyma
m/Ch: Meningeal membranes and choroid plexus
BBB: Brain blood barrier
Iba1: Ionized calcium-binding adapter molecule 1
bDC: Brain dendritic cells
MHC: Major histocompatibility complex
PRR: Pattern recognition receptor
TLR: Toll-like receptors
LPS: Lipopolysacharides
FC: Flow cytometry
IF: Immunofluorescence
MACS: Magnetic-beads based affinity purification
FBS: Fetal bovine serum
PB: Phosphate buffer
PBS: Phosphate buffer saline
SS: Serum saline
ip: Intraperitoneal
PFA: Paraformaldehyde
DAPI: 4′,6-diamino-2-fenilindol
HBSS: Hank’s Balanced Sal Solution
DG: Dentate gyrus
