# Supplementary figures and images for: Senescent accelerated prone 8 (SAMP8) mice as a model of age dependent neuroinflammation
Source: J Neuroinflammation. 2021 Mar 18;18:75. doi: 10.1186/s12974-021-02104-3 (PMC7977588; doi:10.1186/s12974-021-02104-3)

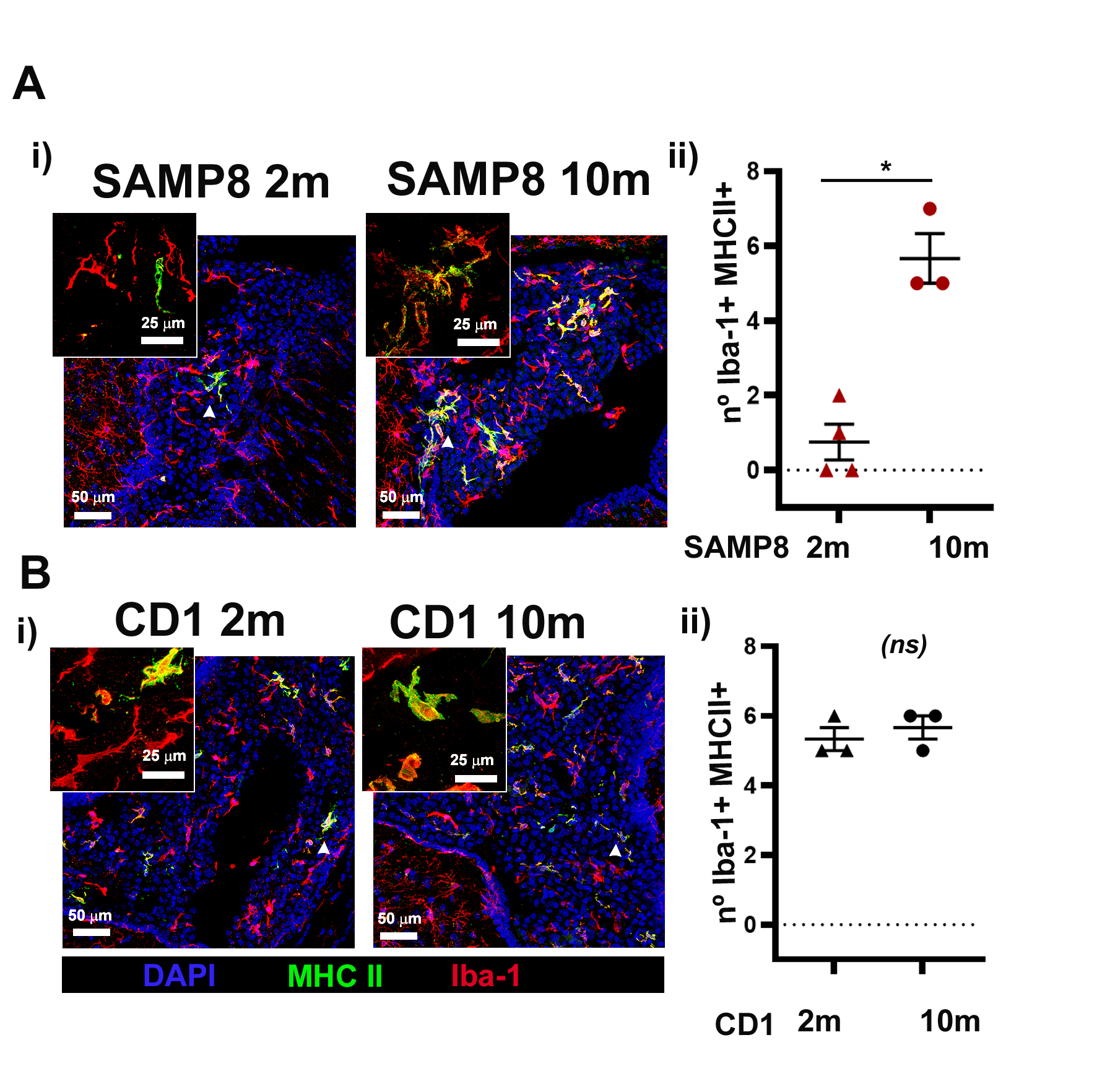

Supplement: Supplementary file 1 — Additional file 1: Supplementary Fig 1S. Evaluation of Iba1+ MHCII+ cells in the choroid plexus of 2 and 10 months old SAMP8 and CD1 mice. Representative Iba1 (red), MHCII (green) and DAPI (blue) staining images of coronal sections of choroid plexus (A) from 2 months (triangles) and 10 months (circles) old SAMP8 in red (B) and 2 months and 10 months CD1 in black. Images were obtained with a Leica TCS SP5 inverted fluorescence confocal microscope, using a 40x and 63x objective (3x digital zoom) for the magnifications. The images are representative of at least three independent experiments using males. Scale bars are included in the images. Panels ii) show analysis of the number of Iba1+ MHCII+ cells over the total of Iba1+ cells. The data show the mean ± SEM (n = 3 to 4 male). * p < 0.05 ; (ns) no significant differences between groups. [file 12974_2021_2104_MOESM1_ESM.tif]

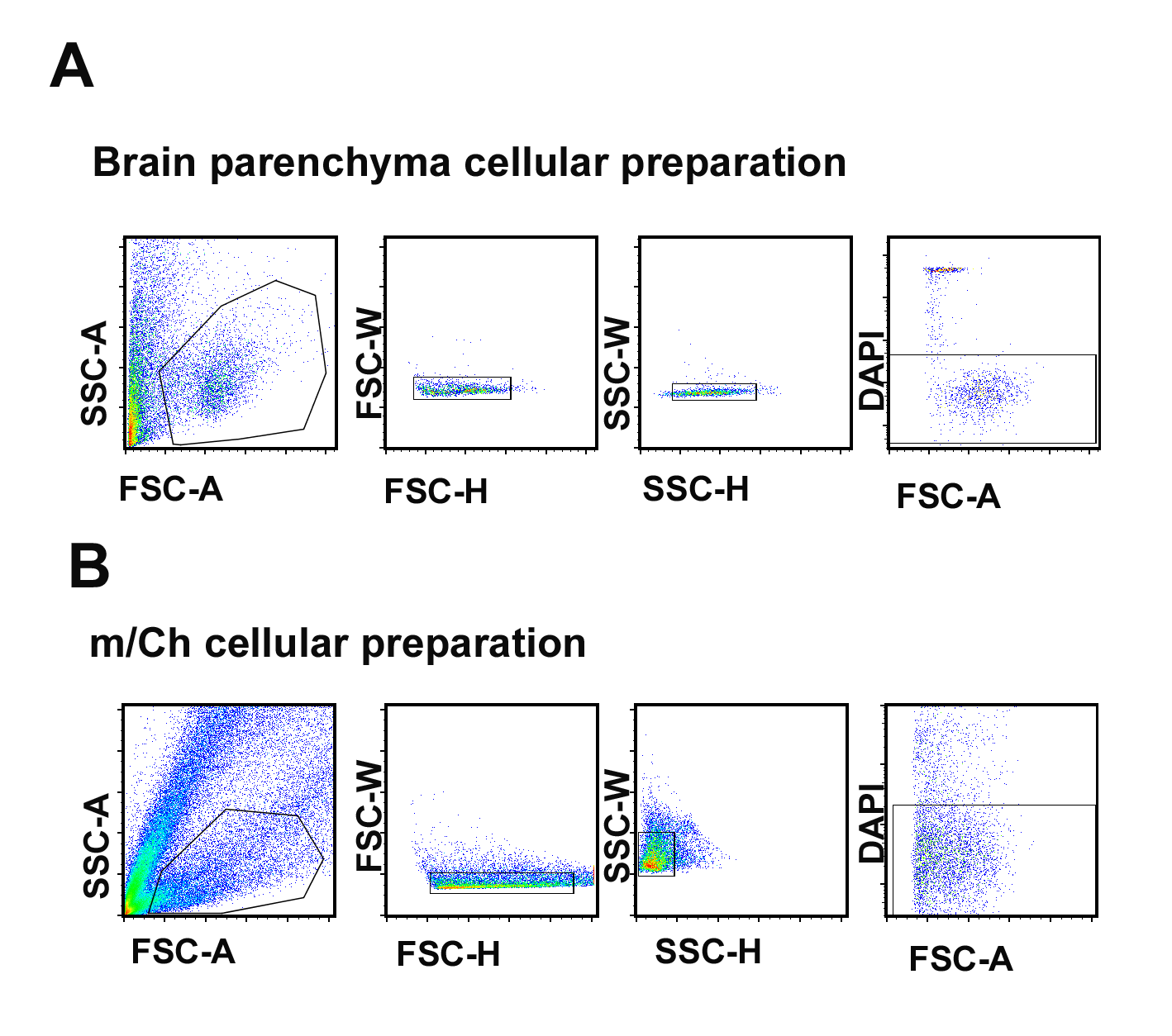

Supplement: Supplementary file 2 — Additional file 2: Supplementary Fig 2S. Gating strategy for flow cytometry analysis. Cells were obtained and labeled as described in Materials and Methods. (A) Brain parenchyma cells without m/Ch (B) m/Ch isolated cells. Before flow cytometry analysis DAPI (5 μg/mL) was added to determine cell viability. Cells were first gated (P1) based on size and complexity (SSC vs FSC). Doublet discrimination was performed by FSC-H vs FSC-W and SSC-H vs SSC-W analyses, and then PI or DAPI negative cells (i.e. live cells) were selected for further analysis. [file 12974_2021_2104_MOESM2_ESM.tif]

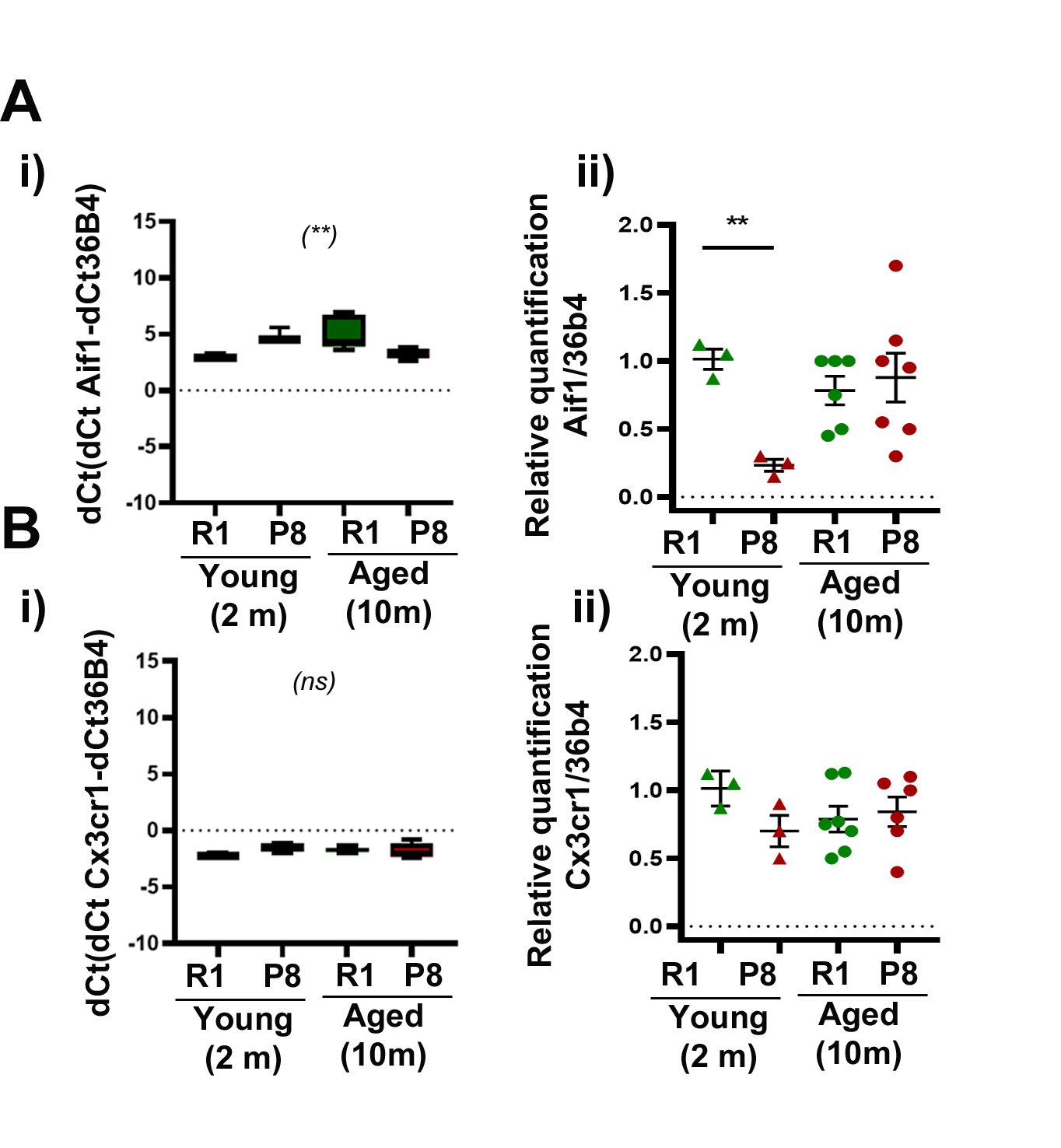

Supplement: Supplementary file 3 — Additional file 3: Supplementary Fig 3S. Aif-1 and Cx3cr1 in CD45+ brain parenchymal cells. Isolation and quantification of BP CD45+ brain cells as in Fig 6. qRT-PCR analysis of (A) Aif-1 gene and (B) Cx3cr1. After extraction of total RNA, cDNA were amplified using SYBR Green Real time PCR methodology using 36b4 as reference gene. (i) Show graphs presented as dCt (Ct gene-Ct 36b4). Lower values of dCt means higher cytokine transcript expression in the sample. (ii) Relative quantification of mRNA expression was referred to average values of 2 m old SAMR1 as control animals that was given a value of 1. The data show the mean ± SEM (n = 3 to 4 males). ** p < 0.01 between specified groups, n ≥ 3 males. [file 12974_2021_2104_MOESM3_ESM.tif]

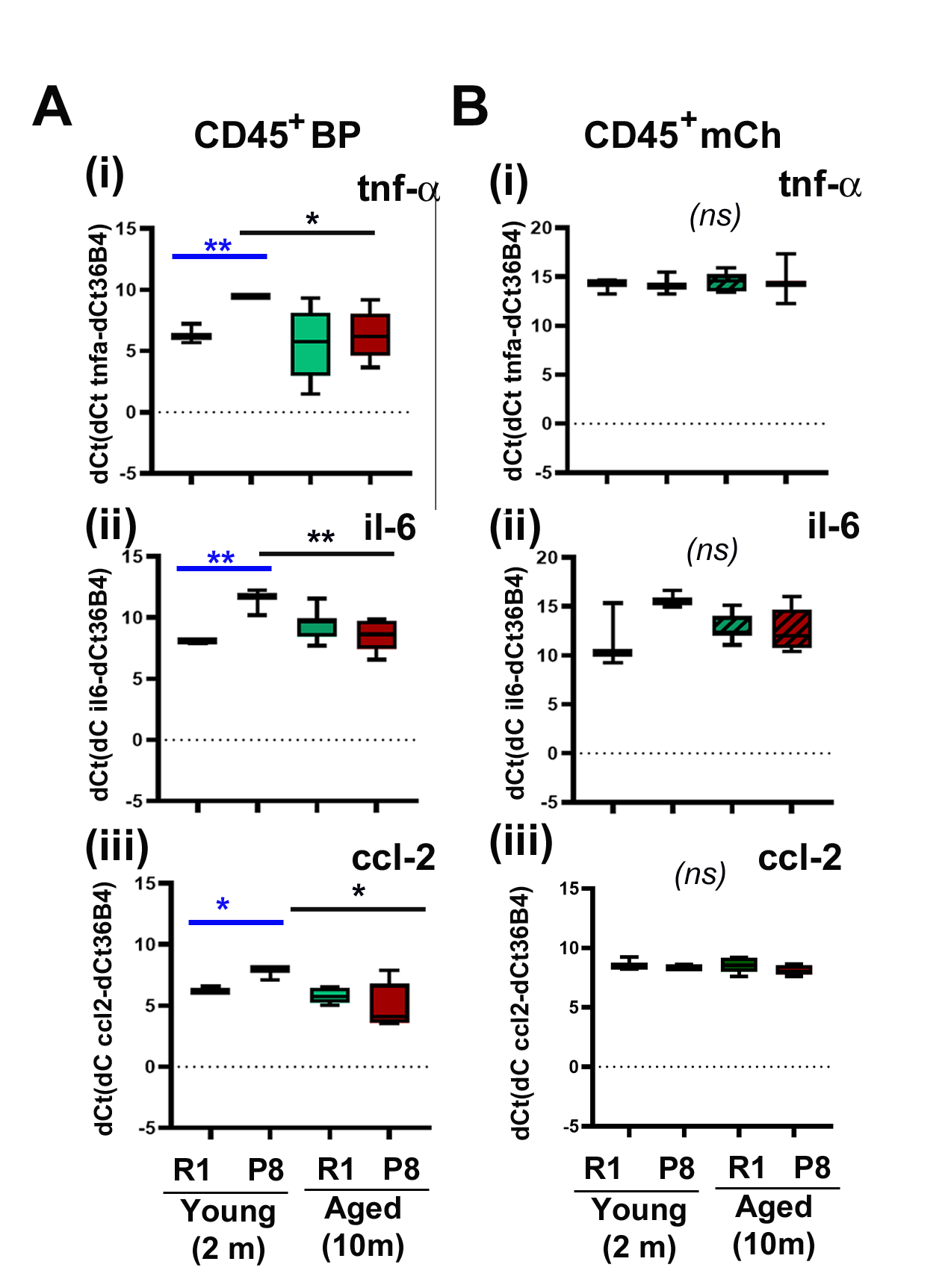

Supplement: Supplementary file 4 — Additional file 4: Supplementary Fig 4S. Tnf-α, Il-6 and Ccl2 expression occurs mainly in aged SAMP8 CD45+ brain parenchymal cells. qRT-PCR analysis of (i) Tnf-α, (ii) Il-6 and (iii) Ccl2 mRNA expression was then quantified by real-time PCR as before. (A) CD45+ brain parenchymal (BP) cells as Fig 6. (B) Dissected choroid plexus and meningeal membranes (m/Ch) were obtained as in Fig 6b. After extraction of total RNA, cDNA were amplified as described in Fig 3s. Data are presented as dCt (Ct gene-Ct 36B4). Lower values of dCt means higher transcript expression of specific mRNA in the sample. The data show the mean ± SEM (n= 3 to 4 males). ** p < 0.01 and *** p < 0.001 between specified groups. [file 12974_2021_2104_MOESM4_ESM.tif]
